# The great time series classification bake off: a review and experimental evaluation of recent algorithmic advances

**DOI:** 10.1007/s10618-016-0483-9

**Published:** 2016-11-23

**Authors:** Anthony Bagnall, Jason Lines, Aaron Bostrom, James Large, Eamonn Keogh

**Affiliations:** 1 School of Computing Sciences, University of East Anglia, Norwich, UK; 2 Computer Science & Engineering Department, University of California, Riverside, Riverside, CA USA

**Keywords:** Time series classification, Shapelets, Elastic distance measures, Time series similarity

## Abstract

In the last 5 years there have been a large number of new time series classification algorithms proposed in the literature. These algorithms have been evaluated on subsets of the 47 data sets in the University of California, Riverside time series classification archive. The archive has recently been expanded to 85 data sets, over half of which have been donated by researchers at the University of East Anglia. Aspects of previous evaluations have made comparisons between algorithms difficult. For example, several different programming languages have been used, experiments involved a single train/test split and some used normalised data whilst others did not. The relaunch of the archive provides a timely opportunity to thoroughly evaluate algorithms on a larger number of datasets. We have implemented 18 recently proposed algorithms in a common Java framework and compared them against two standard benchmark classifiers (and each other) by performing 100 resampling experiments on each of the 85 datasets. We use these results to test several hypotheses relating to whether the algorithms are significantly more accurate than the benchmarks and each other. Our results indicate that only nine of these algorithms are significantly more accurate than both benchmarks and that one classifier, the collective of transformation ensembles, is significantly more accurate than all of the others. All of our experiments and results are reproducible: we release all of our code, results and experimental details and we hope these experiments form the basis for more robust testing of new algorithms in the future.

## Introduction

Time series classification (TSC) problems are differentiated from traditional classification problems because the attributes are ordered. Whether the ordering is by time or not is in fact irrelevant. The important characteristic is that there may be discriminatory features dependent on the ordering. Prior to 2003, there were already at least one hundred papers proposing TSC algorithms. Yet as (Keogh and Kasetty 2003) pointed out that year, in an influential and highly cited paper, the quality of empirical evaluation tended to be very poor relative to the rest of the machine learning community. The majority of TSC algorithms were tested on a single dataset, and the majority of these datasets were synthetic and created by the proposing authors, for the purpose of showcasing their algorithm. The introduction of the University of California, Riverside (UCR) time series classification and clustering repository (Chen et al. 2015) was designed to mitigate these issues. The ready availability of freely available datasets is surely at least partly responsible for the rapid growth in the number of publications proposing time series classification algorithms. Prior to the summer of 2015 over 3000 researchers have downloaded the UCR archive and it has been referenced several hundred times. The repository has contributed to increasing the quality of evaluation of new TSC algorithms. Most experiments involve evaluation on over forty data sets, often with sophisticated significance testing and most authors release source code. This degree of evaluation and reproducibility is generally better than most areas of machine learning and data mining research.

However, there are still some fundamental problems with published TSC research that we aim to address. Firstly, nearly all evaluations are performed on a single train/test split. The original motivation for this was perhaps noble. The original creators of the archive had noted that some authors would occasionally accidently cripple the baseline classifiers. For example, Ge and Smyth (2000) did not z-normalize the time series subsequences for simple Euclidean distance matching, making the Euclidean distance perform essentially randomly on a task were it might be expected to perform perfectly. This example was visually apparent by a careful inspection of the figures in Ge and Smyth (2000), but in a simple table of results such errors would probably never be detected. Thus the single train/test split was designed to anchor the comparisons to a known performance baseline.

Nevertheless, this can lead to over interpreting of results. The majority of machine learning research involves repeated resamples of the data, and we think TSC researchers should follow suit. To illustrate why, consider the following anecdote. We were recently contacted by a researcher who queried our published results for one nearest neighbour (1-NN) dynamic time warping (DTW) on the UCR repository train/test splits. When comparing our accuracy results to theirs, they noticed that in some instances they differed by as much as 6%. Over all the problems there was no significant difference, but clearly we were concerned, as it is a deterministic algorithm. On further investigation, we found out that our data were rounded to six decimal places, their’s to eight. These differences on single splits were caused by small data set sizes and tiny numerical differences (often just a single case classified differently). When resampling, there were no significant differences on individual problems when using six or eight decimal places.

Secondly, there are some anomalies and discrepancies in the UCR data that can bias results. Not all of the data are normalised (e.g. Coffee) and some have been normalised incorrectly (e.g. ECG200). This can make algorithms look better than they really are. For example, most authors cite an error of 17.9% for the Coffee data with 1-NN DTW, and most algorithms easily achieve lower error. However, 17.9% error is for DTW on the non-normalised data. If it is normalised, 1-NN DTW has 0% error, a somewhat harder benchmark to beat. ECG200 has been incorrectly formatted so that the sum of squares of the series can classify the data perfectly. If a classifier uses this feature it should be completely accurate. This will be a further source of bias.

Thirdly, the more frequently a fixed set of problems is used, the greater the danger of overfitting and detecting significant improvement that does not generalise to new problems. We should be constantly seeking new problems and enhancing the repository with new data. This is the only real route to detecting genuine improvement in classifier performance.

Finally, whilst the release of source code is admirable, the fact there is no common framework means it is often very hard to actually use other peoples code. We have reviewed algorithms written in C, C++, Java, Matlab, R and python. Often the code is “research grade”, i.e. designed to achieve the task with little thought to reusability or comprehensibility. There is also the tendency to not provide code that performs model selection, which can lead to suspicions that parameters were selected to minimize test error, thus biasing the results.

To address these problems we have implemented 18 different TSC algorithms in Java, integrated with the WEKA toolkit (Hall et al. 2009). We have applied the following selection criteria for the inclusion of an algorithm. Firstly, the algorithm must have been recently published in a high impact conference or journal. Secondly, it must have been evaluated on some subset of the UCR data. Thirdly, source code must be available. Finally, it must be feasible/within our ability to implement the algorithm in Java. This last criteria lead us to exclude at least two good candidates, described in Sect. 2.7. Often, variants of a classifier are described within the same publication. We have limited each paper to one algorithm and taken the version we consider most representative of the key idea behind the approach.

We have conducted experiments with these algorithms and standard WEKA classifiers on 100 resamples of every data set (each of which is normalised), including the 40 new data sets we have introduced into the archive. In addition to resampling the data sets, we have also conducted extensive model selection for many of the 37 classifiers. Full details of our experimental regime are given in Sect. 3.

We believe that this is one of the largest ever experimental studies conducted in machine learning. We have performed tens of millions of experiments distributed over thousands of nodes of a large high performance computing facility. Nevertheless, the goal of the study is tightly focused and limited. This is meant to act as a springboard for further investigation into a wide range of TSC problems we do not address. Specifically, we assume all series in a problem are equal length, real valued and have no missing values. Classification is offline, and we assume the cases are independent (i.e. we do not perform streaming classification). All series are labelled and all problems involve learning the labels of univariate time series. We are interested in testing hypotheses about the average accuracy of classifiers over a large set of problems. Algorithm efficiency and scalability are of secondary interest at this point. Detecting whether a classifier is on average more accurate than another is only part of the story. Ideally, we would like to know *a priori* which classifier is better for a class of problem or even be able to detect which is best for a specific data set. However, this is beyond the scope of this project.

Our findings are surprising, and a little embarrassing, for two reasons. Firstly, many of the algorithms are in fact no better than our two benchmark classifiers, 1-NN DTW and Rotation Forest. Secondly, of those 9 significantly better than both benchmarks, by far the best classifier is COTE (Bagnall et al. 2015), an algorithm proposed by a subset of the current authors. It is on average over 8% more accurate than either benchmark. Whilst gratifying for us, we fear that this outcome may cause some to question the validity of the study. We have made every effort to faithfully reproduce all algorithms. We have tried to reproduce published results, with varying degrees of success (as described below), and have consulted authors on the implementation where possible. Our results are reproducable, and we welcome all input on improving the code base. We must stress that COTE is by no means the final solution. All of the algorithms we describe may have utility in specific domains, and many are orders of magnitudes faster than COTE. Nevertheless, we believe that it is the responsibility of the designers of an algorithm to demonstrate its worth. We think our benchmarking results will help facilitate an improved understanding of the utility of new algorithms under alternative scenarios.

All of the code is freely accessible from a repository (Bagnall et al., https://bitbucket.org/TonyBagnall/time-series-classification) and detailed results and data sets are available from a dedicated website (Bagnall et al., http://timeseriesclassification.com).

The rest of this paper is structured as follows. In Sect. 2 we review the algorithms we have implemented. In Sect. 3 we describe the data, code structure and experimental design. In Sects. 4 and 5 we present and analyse the results. In Sect. 6 we look in detail at the performance of different algorithms on case study problems and in Sect. 7 we summarise our findings and discuss the future direction.

## Time series classification algorithms

We denote a vector in bold and a matrix in capital bold. A case/instance is a pair $\{\mathbf{x},y\}$ with *m* observations $(x_1, \ldots , x_m)$ (the time series) and discrete class variable *y* with *c* possible values. A list of *n* cases with associated class labels is $\mathbf{T}=(\mathbf{X,y})=((\mathbf{x_1},y_1), \ldots , (\mathbf{x_n},y_n))$. A classifier is a function or mapping from the space of possible inputs to a probability distribution over the class variable values. Time series classification algorithms involve some processing or filtering of the time series values prior or during constructing the classifier. There have been a wide range of approaches to TSC which draw from a large number of diverse research areas. Forming some taxonomy of the algorithms helps in the understanding of the similarity and differences of the various approaches.

There are several alternative ways of grouping algorithms for TSC. We think the most useful involves classifying algorithms by the type of discriminatory features the technique is attempting to find. We classify techniques into the following categories.

1. *Whole series* Two series are compared either as a vector (as with traditional classification) or by a distance measure that uses all the data. Most research effort has been directed at finding techniques that can compensate for small misalignments between series using *elastic* distance measures (Sect. 2.1).
2. *Intervals* Rather than use the whole series, this class of technique selects one or more (phase dependent) intervals of the series. At its simplest, this involves a feature selection of a contiguous subset of attributes. However, as described in Sect. 2.2, better results have been obtained through selecting multiple intervals and using summary measures as features.
3. *Shapelets* A family of algorithms focus on finding short patterns that define a class, but that can appear anywhere in the series. These phase independent patterns are commonly called shapelets (Sect. 2.3). A class is then distinguished by the presence or absence of one or more shapelets somewhere in the whole series.
4. *Dictionary based* Some problems are distinguished by the frequency of repetition of subseries rather than by their presence or absence. Dictionary based methods (Sect. 2.4) form frequency counts of recurring patterns, then build classifiers based on the resulting histograms.
5. *Combinations* A further class of algorithms combines two or more of the above approaches into a single classifier. Two of these techniques are described in Sect. 2.5.
6. *Model based* Model based algorithms fit a generative model to each series then measure similarity between series using similarity between models. Some of the approaches used include fitting auto-regressive models (Bagnall and Janacek 2014; Corduas and Piccolo 2008), hidden Markov models (Smyth 1997) and kernel models (Chen et al. 2013). We do not include any of these approaches in the study for three reasons. Firstly, the techniques are commonly proposed for a task other than classification (e.g. Maharaj 2000) or as part of a larger classification scheme (Bagnall et al. 2015). Secondly, code is unavailable for many of these methods (for example, Chen et al. 2013). Finally, our informal opinion based on experimentation and published results is that many generative model similarity approaches are not competitive for classification of the majority of these datasets. They are more appropriate for long series with unequal length (see Bagnall and Janacek 2014 for more detail).

### Whole series similarity

**Fig. 1:**
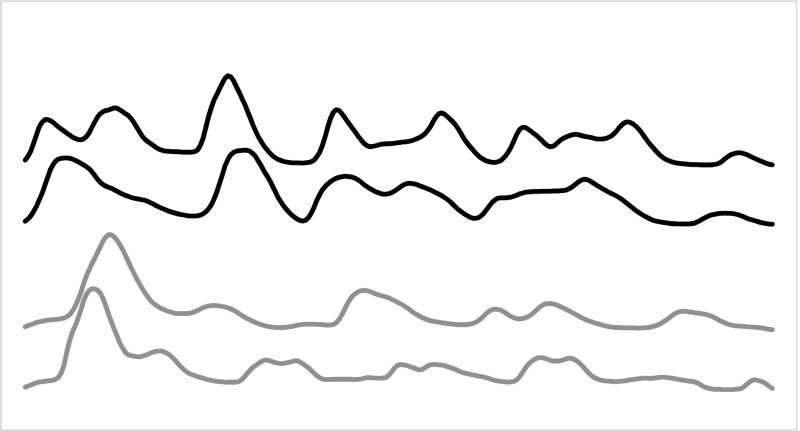
Four cases from two classes of the dataset FiftyWords. The *top two series* show class 30, the *bottom two* class 50. The common pattern is clear, but only detectable with reallignment

Whole series TSC algorithms usually employ a similarity measure between series that quantifies the distance between two series after compensation for localised distortions. These similarity/distance measures are usually employed with a nearest neighbour classifier. Whole series similarity is appropriate when there may be discriminatory features over the whole series, but there may also be some shifting of these features in the time axis. For example, consider the problem FiftyWords. This data set was first used in Rath and Manamatha (2003) and a reformatted version has since become part of the UCR archive. Each case consists of the height profile of one of fifty words taken from the George Washington archive. An example of the data is given in Fig. 1.

Clearly, the whole series is required to distinguish between words, because cases from different classes could differ at any point (e.g. there could be one letter different). Vector based approaches will be confounded by the fact that there may be some dilation in the location of the peaks and troughs. We would expect whole series elastic measures to be the best family of approaches for this sort of problem.

The large majority of time series research in the field of data mining has concentrated on alternative elastic distance measures for measuring the whole series similarity. For TSC, these distance measures are almost exclusively evaluated using a one nearest neighbour (1-NN) classifier. The standard benchmark elastic distance measure is dynamic time warping (DTW).

#### Dynamic time warping

Suppose we want to measure the distance between two series, ${\mathbf {a}}=(a_1,a_2,\ldots ,a_m)$ and ${\mathbf {b}}=(b_1,b_2,\ldots ,b_m)$. Let $M({\mathbf {a}},{\mathbf {b}})$ be the $m \times m$ pointwise distance matrix between ${\mathbf {a}}$ and ${\mathbf {b}}$, where $M_{i,j}= (a_i-b_j)^2$. A warping path

$$\begin{aligned} P=((e_1,f_1),(e_2,f_2),\ldots ,(e_s,f_s)) \end{aligned}$$

is a series of points (i.e. pairs of indexes) that define a traversal of matrix *M*. So, for example, the Euclidean distance $d_E(\mathbf {a,b})=\sum _{i=1}^m (a_i-b_i)^2$ is the path along the diagonal of *M*. A valid warping path must satisfy the conditions $(e_1,f_1)=(1,1)$ and $(e_s,f_s)=(m,m)$ and that $0 \le e_{i+1}-e_{i} \le 1$ and $0 \le f_{i+1}- f_i \le 1$ for all $i < m$. The DTW distance between series is the path through *M* that minimizes the total distance, subject to constraints on the amount of warping allowed. Let $p_i=M_{{e_i},{f_i}}$ be the distance between elements at position $e_i$ of ${\mathbf {a}}$ and at position $f_i$ of ${\mathbf {b}}$ for the $i\mathrm{th}$ pair of points in a proposed warping path *P*. The distance for any path *P* is

$$\begin{aligned} D_P({\mathbf {a}},{\mathbf {b}}) =\sum _{i=1}^s p_i. \end{aligned}$$

If ${\mathcal {P}}$ is the space of all possible paths, the DTW path $P^*$ is the path that has the minimum distance, i.e.

$$\begin{aligned} P^* = \min _{P \in {\mathcal {P}}}(D_P({\mathbf {a}},{\mathbf {b}})). \end{aligned}$$

The optimal path $P^*$ can be found exactly through a dynamic programming formulation. This can be a time consuming operation, and it is common to put a restriction on the amount of warping allowed. This restriction is equivalent to putting a maximum allowable distance between any pairs of indexes in a proposed path. If the warping window, *r*, is the proportion of warping allowed, then the optimal path is constrained so that

$$\begin{aligned} |e_i-f_i| \le r\cdot m \quad \forall (e_i,f_i) \in P^*. \end{aligned}$$

It has been shown that setting *r* through cross validation to maximize training accuracy, as proposed in Ratanamahatana and Keogh (2005), significantly increases accuracy on unseen data (Lines and Bagnall 2015). Numerous speed ups for DTW have also been described in the literature (Rakthanmanon et al. 2013).

Many alternatives to DTW have been proposed. Distance measures include edit distance with real penalty (ERP) (Chen and Ng 2004) and longest common subsequence (LCSS) (Hirschberg 1977). Other approaches use differences between the first order differences of the whole series. However, in 2008, Ding et al. (2008) and Wang et al. (2013) evaluated eight different distance measures on 38 data sets and found none significantly better than DTW. Since then, many more elastic measures have been proposed. We assess the three most prominent that meet our selection criteria as described in the introduction.

#### Weighted DTW (WDTW) (Jeong et al. 2011)

Jeong et al. describe WDTW (Jeong et al. 2011), which adds a multiplicative weight penalty based on the warping distance between points in the warping path. It favours reduced warping, and is a smooth alternative to the cutoff point approach of using a warping window. When creating the distance matrix *M*, a weight penalty $w_{|i-j|}$ for a warping distance of $|i-j|$ is applied, so that

$$\begin{aligned} M_{i,j}= w_{|i-j|} (a_i-b_j)^2. \end{aligned}$$

A logistic weight function is used, so that a warping of *a* places imposes a weighting of

$$\begin{aligned} w(a)=\frac{w_{max}}{1+e^{-g\cdot (a-m/2)}}, \end{aligned}$$

where $w_{max}$ is an upper bound on the weight (set to 1), *m* is the series length and *g* is a parameter that controls the penalty level for large warpings. The larger *g* is, the greater the penalty for warping.

#### Time warp edit (TWE) (Marteau 2009)

Marteau proposes the TWE distance (Marteau 2009), an elastic distance metric that includes characteristics from both LCSS and DTW. It allows warping in the time axis and combines the edit distance with L$_p$-norms. The warping is controlled by a *stiffness* parameter, $\nu$. Stiffness enforces a multiplicative penalty on the distance between matched points in a manner similar to WDTW. A penalty value $\lambda$ is applied when sequences do not match (Algorithm 1).
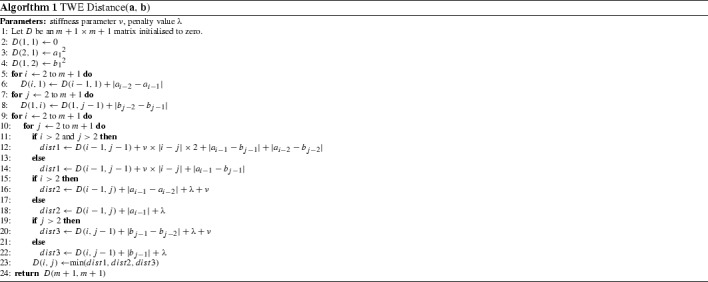


#### Move–split–merge (MSM) (Stefan et al. 2013)

Stefan et al. (2013) present MSM distance (Algorithm 2), a metric that is conceptually similar to other edit distance-based approaches, where similarity is calculated by using a set of operations to transform a given series into a target series. Move is synonymous with a substitute operation, where one value is replaced by another. The split operation inserts an identical copy of a value immediately after itself, and the merge operation is used to delete a value if it directly follows an identical value.
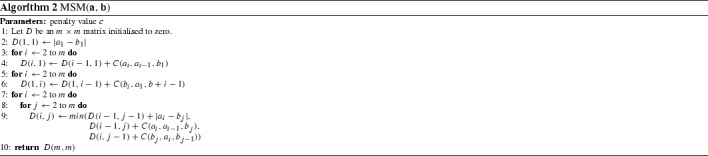

$$\begin{aligned} C(a_i,a_{i-1},b_j) = \left\{ \begin{array}{l} c~\mathbf{if}~a_{i-1} \le a_i \le b_j ~\mathbf{or}~a_{i-1} \ge a_i \ge b_j \\ c+min(|a_i-a_{i-1}|,|a_i-b_j|)~\mathbf{otherwise}. \end{array} \right. \end{aligned}$$

We have implemented WDTW, TWE, MSM and other commonly used time domain distance measures (such as LCSS and ERP). They are available in the package elastic_distance_measures in the code base (Bagnall et al., https://bitbucket.org/TonyBagnall/time-series-classification). We have generated results that are not significantly different to those published when using these distances with 1-NN. In Lines and Bagnall (2015) it was shown that there is no significant difference between 1-NN with DTW and with WDTW, TWE or MSM on a set of 72 problems using a single train/test split. In Sect. 4 we revisit this result with more data and resamples rather than a train/test split.

There are a group of algorithms that are based on whole series similarity of the first order differences of the series,

$$\begin{aligned} a^{\prime }_i = a_i-a_{i+1}, \quad i=1 \ldots m-1, \end{aligned}$$

which we refer to as *diff*. Various methods that have used just the differences have been described (Jeong et al. 2011), but the most successful approaches combine distance in the time domain and the difference domain.

#### Complexity invariant distance (CID) (Batista et al. 2014)

Batista et al. (2014) describe a means of weighting a distance measure to compensate for differences in the complexity in the two series being compared. 
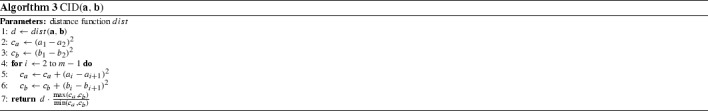
 Any measure of complexity can be used, but Batista et al. recommend the simple expedient of using the sum of squares of the first differences (see Algorithm 3). CID is evaluated with ED and DTW. We use the latter measure. For consistency with the published algorithm, window size for DTW is set using cross validation of DTW distance (rather than CID).

#### Derivative DTW ($\hbox {DD}_{\textit{DTW}}$) (Górecki and Łuczak 2013)

Górecki and Łuczak (2013) describe an approach for using a weighted combination of raw series and first-order differences for NN classification with either the Euclidean distance or full-window DTW. They find the DTW distance between two series and the two differenced series. These two distances are then combined using a weighting parameter $\alpha$ (See Algorithm 4). Parameter $\alpha$ is found during training through a leave-one-out cross-validation on the training data. This search is relatively efficient as different parameter values can be assessed using pre-computed distances.
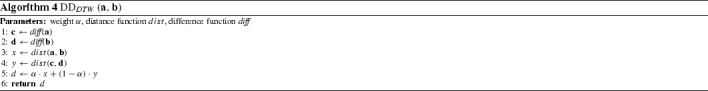


An optimisation to reduce the search space of possible parameter values is proposed in Górecki and Łuczak (2013). However, we could not recreate their results using this optimisation. We found that if we searched through all values of $\alpha$ in the range of [0, 1] in increments of 0.01, we were able to recreate the results exactly. Testing is then performed with a 1-NN classifier using the combined distance function given in Algorithm 4.

#### Derivative transform distance ($\hbox {DTD}_C$) (Górecki and Łuczak 2014)

Górecki and Łuczak proposed an extension of $\hbox {DD}_{\textit{DTW}}$ that uses DTW in conjunction with transforms and derivatives (Górecki and Łuczak 2014). They propose and evaluate combining $\hbox {DD}_{\textit{DTW}}$ with distances on data transformed with the sin, cosine and Hilbert transform. We implement the cosine version (see Algorithm 5), where function *cos* transforms a series $\mathbf{a}$ into $\mathbf{c}$ using the formula

$$\begin{aligned} c_i = \sum _{j=1}^m a_j \cos \left( \frac{\varPi }{2} \left( j - \frac{1}{2} \right) (i-1) \right) \quad i=1 \ldots m. \end{aligned}$$

The two parameters $\alpha$ and $\beta$ are found through a grid search. 
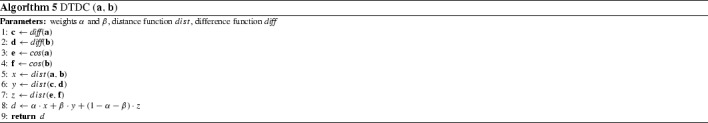


$\hbox {DD}_{\textit{DTW}}$ was evaluated on single train/test splits of 20 UCR datasets in Górecki and Łuczak (2013) and both $\hbox {DD}_{\textit{DTW}}$ and $\hbox {DTD}_C$ were evaluated on 47 in Górecki and Łuczak (2014), $\hbox {CID}_{\textit{DTW}}$ on 43 datasets and $\hbox {DTD}_C$ on 47. We can recreate results that are not significantly different to those published for all three algorithms. All papers claim superiority to DTW. The small sample size for $\hbox {DD}_{\textit{DTW}}$ makes this claim debatable, but the published results for $\hbox {CID}_{\textit{DTW}}$ and $\hbox {DTD}_C$ are both significantly better than DTW. On published results, $\hbox {DTD}_C$ is significantly more accurate than $\hbox {CID}_{\textit{DTW}}$ and $\hbox {CID}_{\textit{DTW}}$ is significantly better than $\hbox {DD}_{\textit{DTW}}$.

#### Elastic ensemble (EE) (Lines and Bagnall 2015)

The EE is a combination of 11 nearest neighbour (NN) classifiers that use whole series elastic distance measures in the time domain and with first order derivatives. The 11 classifiers in EE are 1-NN with: Euclidean distance; full window DTW; DTW with window size set through cross validation; derivative DTW with full window; derivative DTW with window set through cross validation; weighted DTW; derivative weighted DTW; LCSS; ERP; time warp edit distance (Marteau 2009), and the move–split–merge distance metric (Stefan et al. 2013). Lines and Bagnall (2015) show that none of the individual components of EE significantly outperforms DTW with window set through cross validation. They then demonstrate that an ensemble of these 11 whole series elastic measures that combines the predictions of 1-NN classifiers using a voting scheme that weights according to cross-validation training set accuracy is significantly more accurate than any single component, including DTW.

### Phase dependent intervals

**Fig. 2:**
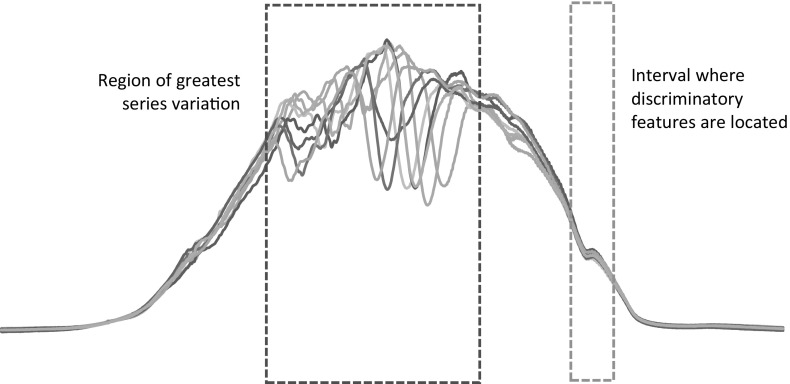
An example dataset where interval methods should do well. The noise in the early part of the series may confound whole series methods

A family of algorithms derive features from intervals of each series. For a series of length *m*, there are $m(m-1)/2$ possible contiguous intervals. The type of problem that will benefit from selecting intervals rather than using whole series is likely to involve long series with phase dependent discriminatory subseries and regions of noise that could confound the classifier. For example, consider the problem SmallKitchenAppliances, first used in Lines and Bagnall (2015) and derived from a UK study into power usage in the home (Energy Saving Trust 2012). The SmallKitchenAppliances problem involves classifying an electric device as either a kettle, microwave or toaster based on a daily usage profile. The data is recorded every 2 min, hence a case consists of 720 measurements. The pattern of usage is obviously discriminatory, but so is the time of usage. For example, toasters are used more frequently in the morning, microwaves in the evening. Each device is used between 1 and 10 times per day, so there are large areas of redundant information in each instance. An interval based approach that uses numerous intervals should be able to mitigate against this confounding effect. Normalising makes the pattern of usage more important, as it distorts the actual electricity usage. At their simplest, interval based methods are simply attribute selection technqiues. Consider the spectral classification shown in Fig. 2. The data are light readings across a wide spectra. The discriminatory features are at the infrared region of the spectrum. However, ambient conditions cause huge variation in the visible light range which is independent of class. This variation may swamp a whole series distance measure and confound a traditional classifier. If we just use the correct interval, classification will be much more accurate. The problem then is finding the best interval. Rather than search for a fixed number of intervals using the training data, TSC algorithms generate many different random intervals and classifiers on each one, ensembling the resulting predictions.

The two key decisions about using random intervals to classify are, firstly, how to deal with the fact there are so many possible intervals and secondly, what to actually do with each interval once selected. Rodríguez et al. (2005) were the first to adopt an interval based approach. They address the first issue by using only intervals of lengths equal to powers of two and the second by calculating binary features over each intervals based on threshold rules on the interval mean and standard deviation. A support vector machine is then trained on this transformed feature set. This algorithm was a precursor to three recently proposed interval based classifiers that we have implemented.

#### Time series forest (TSF) (Deng et al. 2013)

Deng et al. (2013) overcome the problem of the huge interval feature space by employing a random forest approach, using summary statistics of each interval as features. Training a single tree involves selecting $\sqrt{m}$ random intervals, generating the mean, standard deviation and slope of the random intervals for every series then creating and training a tree on the resulting $3\sqrt{m}$ features. Classification is by a majority vote of all the trees in the ensemble.
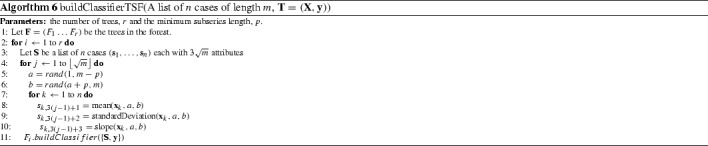


The classification tree has two bespoke characteristics. Firstly, rather than evaluate all possible split points to find the best information gain, a fixed number of evaluation points is pre-defined. We assume this is an expedient to make the classifier faster, as it removes the need to sort the cases by each attribute value. Secondly, a refined splitting criteria to choose between features with equal information gain is introduced. This is defined as the distance between the splitting margin and the closest case. The intuition behind the idea is that if two splits have equal entropy gain, then the split that is furthest from the nearest case should be preferred. This measure would have no value if all possible intervals were evaluated because by definition the split points are taken as equi-distant between cases. We experimented with including these two features, but found the effect on accuracy was, if anything, negative. We found the computational overhead of evaluating all split points acceptable, hence we had no need to include the margin based tie breaker. We used the built in WEKA RandomTree classifier (which is the basis for the WEKA RandomForest classifier) with default parameters. This means there is no limit to the depth of the tree nor a minimum number of cases per leaf node. A more formal description is given in Algorithm 6.

#### Time series bag of features (TSBF) (Baydogan et al. 2013)

Time series bag of features (TSBF) is an extension of TSF that has multiple stages. The first stage involves generating a new classification problem that involves creating new instances from subseries of the full data. The second stage forms class probability estimates for each subseries. The third stage constructs a bag of features for each original instance from these probabilities. Finally a random forest classifier is built on the bag of features representation. Algorithm 7 gives a pseudo-code overview, modularised for clarity. TSBF can be summarised as follows.
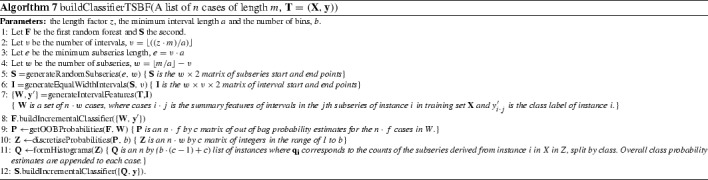


*Stage 1: Generate a subseries classification problem.*

1. Select *w* subseries start and end points (line 5). These are the same for each of the full series. Then, for every series, repeat the following steps
  - for each of the *w* subseries, take *v* equal width intervals (line 6) and calculate the mean, standard deviation and slope (line 7).
  - concatenate these features and the full subseries stats to form a new case with $w\cdot v+3$ attributes and class label of the original series (line 7).

*Stage 2: Produce class probability estimates for each subseries.*

1. Train a random forest on the new subseries dataset $\mathbf{W}$ (line 8). $\mathbf{W}$ contains $n\cdot w$ cases, each with $w\cdot v+3$ attributes. The number of trees in the random forest is determined by incrementally adding trees in groups of 50 until the out of bag error stops decreasing.
2. Find the random forest out of bag estimates of the class probabilities for each subseries (line 9).

*Stage 3: Recombine class probabilities and form a bag of patterns for each series.*

1. Discretise the class probability estimates for each subseries into *b* equal width bins (line 10).
2. Bag together these discretised probabilities for each original series, ignoring the last class (line 11). If there are *c* classes, each instance will have $w \cdot (c-1)$ attributes.
3. Add on the relative frequency of each predicted class (line 11).

*Stage 4: Build the final random forest classifier (line 12).*

New cases are classified by following the same stages of transformation and internal classification. The number of subseries and the number of intervals are determined by a parameter, *z*. Training involves searching possible values of *z* for the one that minimizes the out of bag error for the final classifier. Other parameters are fixed for all experiments. These are the minimum interval length ($a=5$), the number of bins for the discretisation ($b=10$), the maximum number of trees in the forest (1000), the number of trees to add at each step (50) and the number of repetitions (10).

#### Learned pattern similarity (LPS) (Baydogan and Runger 2016)

LPS was developed by the same research group as TSF and TSBF at Arizona State University. It is also based on intervals, but the main difference is that subseries become attributes rather than cases. Like TSBF, building the final model involves first building an internal predictive model. However, LPS creates an internal regression model rather than a classification model. The internal model is designed to detect correlations between subseries, and in this sense is an approximation of an autocorellation function. LPS selects random subseries. For each location, the subseries in the original data are concatenated to form a new attribute. The internal model selects a random attribute as the response variable then constructs a regression tree. A collection of these regression trees are processed to form a new set of instances based on the counts of the number of subseries at each leaf node of each tree. Algorithm 8 describes the process. LPS can be summarised as follows:
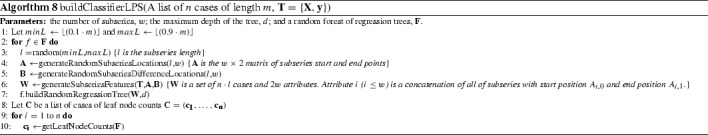


*Stage 1: Construct an ensemble of r regression trees.*

1. Randomly select a segment length *l* (line 3)
2. Select *w* segments of length *l* from each series storing the locations in matrix $\mathbf{A}$ (line 4).
3. Select *w* segments of length *l* from each difference series storing the locations in matrix $\mathbf{B}$ (line 5).
4. Generate the $n \cdot l$ cases each with 2*w* attributes and store in $\mathbf{W}$ (line 6).
5. Choose a random column from $\mathbf{W}$ as the response variable then build a random regression tree (i.e. a tree that only considers one randomly selected attribute at each level) with maximum depth of *d* (line 7).

*Stage 2: Form a count distribution over each tree’s leaf node.*

1. For each case $\mathbf{x}$ in the original data, get the number of rows of $\mathbf{W}$ that reside in each leaf node for all cases originating from $\mathbf{x}$.
2. Concatenate these counts to form a new instance. Thus if every tree had *t* terminal nodes, the new case would have $r\cdot t$ features. In reality, each tree will have a different number of terminal nodes.

Classification of new cases is based on a 1-NN classification on these concatenated leaf node counts.

There are two versions of LPS available, both of which aim to avoid the problem of generating all possible subseries. The R version with embedded C functions creates the randomly selected attribute at Stage 1 on the fly at each level of the tree. This avoids the need to generate all possible subseries, but requires a bespoke tree. The second implementation (in Matlab) fixes the number of subseries to randomly select for each tree. Experiments suggest there is little difference in accuracy between the two approaches. We adopt the latter algorithm because it allows us to use the WEKA RandomRegressionTree algorithm, thus simplifying the code and reducing the likelihood of bugs.

**Fig. 3:**
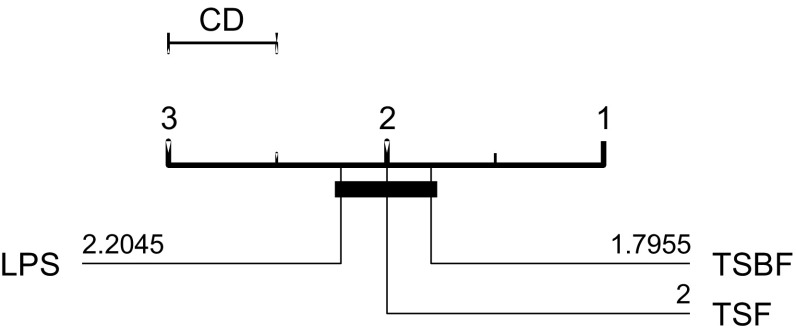
Average ranks of published results for TSF, LPS and TSBF in a critical difference diagram (explained in detail in Sect. 3)

TSF and TSBF were evaluated on the original 46 UCR problems, LPS on an extended set of 75 data sets first used in Lines and Bagnall (2015) (with the standard single train/test splits). Figure 3 shows the ranks of the published results for the problem sets they have in common. Although TSBF has the highest average rank, there is no significant difference between the classifiers at the 5% level. Pairwise comparisons yield no significant difference between the three.

All three algorithms are stochastic, and our implementations are not identical, so there are bound to be variations between our results and those found with the original software. Our implementation of TSF has higher accuracy on 21 of the 44 datasets, worse on 23. The mean difference in accuracy is less than 1%. There is no significant difference in means (at the 5% level) with a rank sum test or a binomial test.

Not all of the 75 datasets LPS used are directly comparable to those in the new archive. This is because all of the new archive have been normalised, whereas many of the data proposed in Lines and Bagnall (2015) are not normalised. Hence we restrict our attention to the original UCR datasets. Our LPS classifier has higher accuracy on 20 of the 44 datasets and worse on 23. The mean difference in accuracy is less than 0.02%. Our results are not significantly different to those published when tested with a rank sum test and a binomial test.

Our TSBF results are significantly worse than those published. Our TSBF classifier has higher accuracy on 9 of the 44 datasets, worse on 34. The mean difference is just over 1%. There is no obvious reason for this discrepancy. TSBF is a complex algorithm, and it is possible there is a mistake in our implementation, but through our best debugging efforts and consultation with the algorithm author we were not able to find one. It may be caused by a difference in the random forest implementations of R and WEKA or by an alternative model selection method.

### Phase independent shapelets

**Fig. 4:**
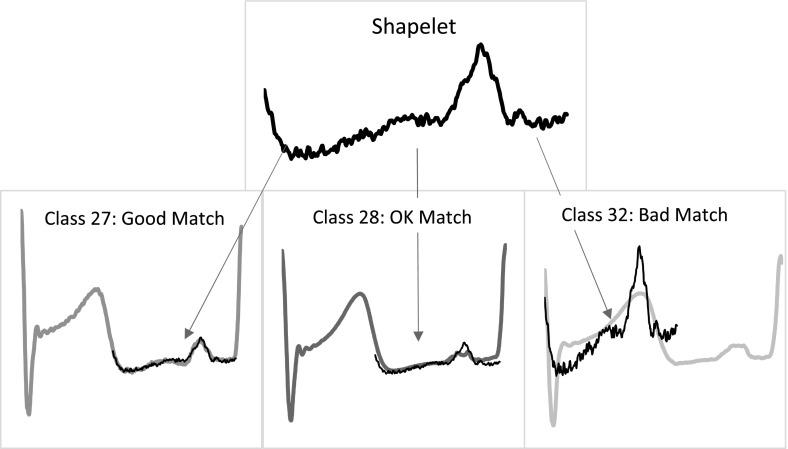
Example matching between a shapelet and three series of different classes from NonInvasiveFetalECGThorax2. The scale of the shapelet is different on each series to reflect that distance is measured with normalised subseries

The third scenario we consider is when one or more patterns within a series define a class, but the actual location of this pattern is irrelevant. For example, a class of abnormal ECG measurement may be characterised by an unusual pattern that only occurs occasionally at any point during the measurement. Shapelets are subseries that capture this type of characteristic. They allow for the detection of phase-independent localised similarity between series within the same class. Figure 4 shows some example series from the dataset NonInvasiveFetalECGThorax2. The data series are 750 points long and there are 42 classes. Each series represents a single fetal heartbeat, so a single anomaly will differentiate between one class and the others, but this may occur at different locations. Understandably, full series methods perform relatively poorly on this data. Interval based methods may do better because they can remove some of the noise caused by the length of series, but they still rely on the discriminatory features occurring in approximately the same location. Figure 4 also displays an example shapelet matched to three NonInvasiveFetalECGThorax2 series. This shapelet provides a good match for class 27 but a poor match for class 32.

The original shapelet algorithm by Ye and Keogh (2011) finds shapelets through enumerating all possible candidates, then uses the best shapelet as the splitting criterion at each node of a decision tree. Two characteristics of the Ye algorithm are that, firstly, shapelet enumeration is slow ($O(n^2m^4)$) and secondly, using shapelets embedded in a decision tree does not lead to particularly accurate classifiers. This has lead to the three recent advances in using shapelets for classification.

#### Fast shapelets (FS) (Rakthanmanon and Keogh 2013)

Rakthanmanon and Keogh (2013) propose an extension of the decision tree shapelet approach (Ye and Keogh 2011; Mueen et al. 2011) that speeds up shapelet discovery. Instead of a full enumerative search at each node, the fast shapelets algorithm discretises and approximates the shapelets using a symbolic aggregate approximation (SAX) (Lin et al. 2007). SAX is a method for converting series to strings that reduces the dimension of a series through piecewise aggregate approximation (PAA) (Chakrabarti et al. 2002), then discretises the (normalised) series into bins formed from equal probability areas of the normal distribution.

The FS algorithm forms a dictionary of SAX words for each possible shapelet length. The dimensionality of the SAX dictionary is reduced through masking randomly selected letters (random projection). Multiple random projections are performed, and a frequency count histogram is built for each class. A score for each SAX word can be calculated based on how well these frequency tables discriminate between classes. The *k*-best SAX words are selected then mapped back to the original shapelets, which are assessed using information gain in a way identical to that used in Ye and Keogh (2011). Algorithm 9 gives a modular overview.
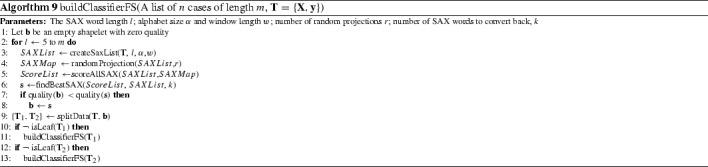


#### Shapelet transform (ST) (Hills et al. 2014; Bostrom and Bagnall 2015)

Hills et al. (2014) propose a shapelet transformation that separates the shapelet discovery from the classifier by finding the top *k* shapelets on a single run (in contrast to the decision tree, which searches for the best shapelet at each node). The shapelets are used to transform the data, where each attribute in the new dataset represents the distance of a series to one of the shapelets. We use the most recent version of this transform (Bostrom and Bagnall 2015) that balances the number of shapelets per class and evaluates each shapelet on how well it discriminates just one class. Algorithm 10 describes the enumerative process of finding *k* / *c* shapelets for each class, where *c* is the number of classes. For each series, all candidate shapelets of all permissable lengths are assessed for their discriminatory power. This is done in two stages. First, we find the distance between a shapelet and all series in the data. The distance between a shapelet $\mathbf{a}$ of length *l* and a series $\mathbf{t}$ of length $m>l$ is found by finding the minimum Euclidean distance between the shapelet and all $m-l+1$ windows in $\mathbf{t}$ (function findDistances in line 10). Second, the *n* distances stored in list $\mathbf{d}$ are used to measure how well the shapelet discriminates between series (using the information gain) of class *i* and those not of class *i* (line 11 function assessCandidate). The candidate list *r* is sorted then overlapping candidates are removed (line 14 removeSelfSimilar). The top *k* / *c* shapelets for each class are retained.
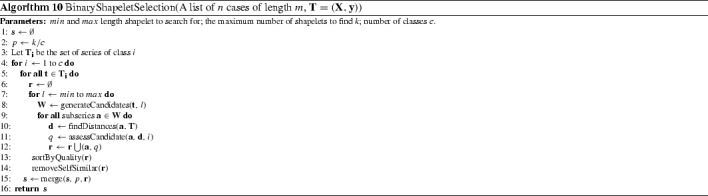


The algorithm described in Algorithm 10 creates a set of shapelets. These shapelets are used to create a new dataset, where an attribute represents the distance between each series and a shapelet. Following Bagnall et al. (2015) and Bostrom and Bagnall (2015) we construct a classifier from this dataset using a weighted ensemble of standard classifiers. We include *k*-Nearest Neighbour (where *k* is set through cross validation), Naive Bayes, C4.5 decision tree, Support Vector Machines with linear and quadratic basis function kernels, Random Forest (with 500 trees), Rotation Forest (with 50 trees) and a Bayesian network. Each classifier is assigned a weight based on the cross validation training accuracy, and new data (after transformation) are classified with a weighted vote. With the exception of *k*-NN, we do not optimise parameter settings for these classifiers via cross validation.

#### Learned shapelets (LS) (Grabocka et al. 2014)

Grabocka et al. (2014) describe a shapelet discovery algorithm that adopts a heuristic gradient descent shapelet search procedure rather than enumeration. LS finds *k* shapelets that, unlike FS and ST, are not restricted to being subseries in the training data. The *k* shapelets are initialised through a *k*-means clustering of candidates from the training data. The objective function for the optimisation process is a logistic loss function (with regularization term) *L* based on a logistic regression model for each class. The algorithm jointly learns the weights for the regression $\mathbf{W}$, and the shapelets $\mathbf{S}$ in a two stage iterative process to produce a final logistic regression model.
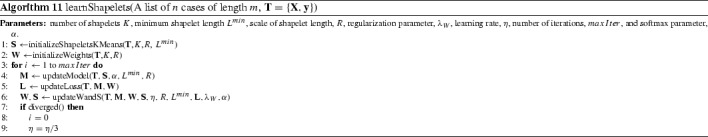


Algorithm 11 gives a high level view of the algorithm. LS restricts the search to shapelets of length $\{L^{min},2L^{min},\ldots ,RL^{min}\}.$ A check is performed at certain intervals as to whether divergence has occurred (line 7). This is defined as a train set error of 1 or infinite loss. The check is performed when half the number of allowed iterations is complete. This criteria meant that for some problems, LS never terminated during model selection. Hence we limited the the algorithm to a maximum of five restarts.

**Fig. 5:**
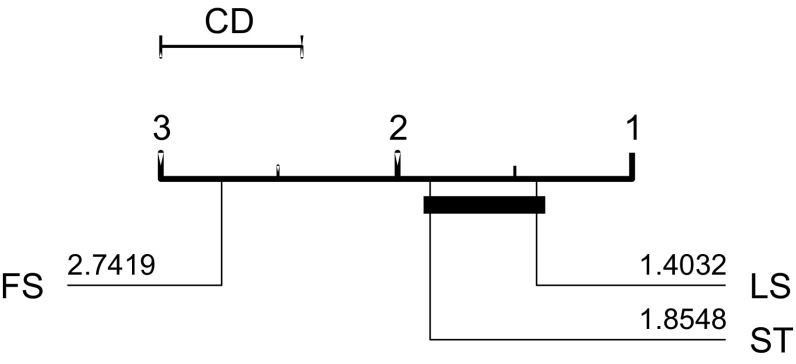
Average ranks of the published results for three shapelet algorithms Fast Shapelets (FS), Shapelet Transform (ST) and Learned Shapelets (LS) on the 33 datasets they have in common

FS, LS and ST were evaluated on 33, 45 and 75 data sets respectively. The published results for FS are significantly worse than those for LS and ST (see Fig. 5). There is no significant difference between the LS and ST published results.

We can reproduce results that are not significantly different to FS and ST. We can reproduce the output of the base code released for LS but are unable to reproduce the actual published results. On average, our results are 1% worse than those published, with the published results better on 26 datasets and worse on 7. The author of LS has contributed several important refinements to the code and is looking to further bridge this gap.

### Dictionary based classifiers

**Fig. 6:**
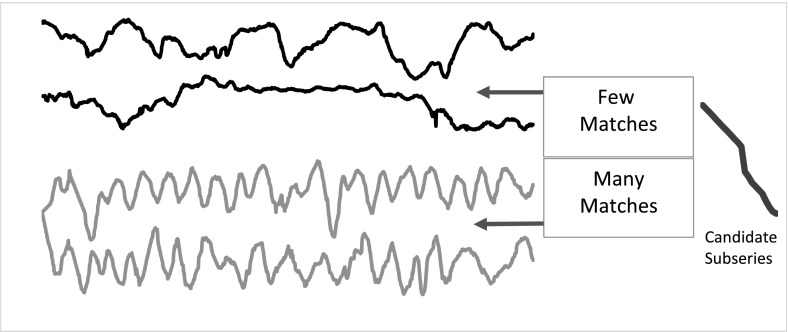
An example of the need for detecting recurring patterns rather than unique patterns from WormsTwoClass. The *top two series* are the motion of normal worms, the *bottom two* mutant worms. The candidate subseries would not necessarily be a good shapelet, because there are close matches in both mutant and non mutant series

Shapelet classifiers can find a single phase independent pattern that differentiates classes. However, if it is the relative frequency of patterns that distinguishes the classes, the shapelet approach will fail, because it looks for the closest single match in each series, not the number of repetitions. Consider the problem WormsTwoClass, which involves detecting whether a *Caenorhabditis elegans* is a mutant based on its motion (first used in Bagnall et al. 2015 and derived from Yemini et al. 2013) (Fig. 6).

Mutants are characterised by the fact they make repeated unusual moves that normal worms rarely make. However, because the series are relatively long it is likely the normal worms may make the unusual move at least once. This will confound shapelet algorithms. Dictionary approaches address this scenario by forming a frequency count of repeated patterns. To do this they approximate and reduce the dimensionality of series by transforming them into representative words, then basing similarity on comparing the distribution of words. The core process of dictionary approaches involves forming words by passing a sliding window, length *w*, over each series, approximating each window to produce *l* values, and then discretising these values by assigning each a symbol from an alphabet of size $\alpha$.

#### Bag of patterns (BOP) (Lin et al. 2012)

BOP is a dictionary classifier built on the SAX (Lin et al. 2007). BOP works by applying SAX to each window to form a word. If consecutive windows produce identical words, then only the first of that run is recorded. This is included to avoid over counting trivial matches. The distribution of words over a series forms a count histogram. To classify new samples, the same transform is applied to the new series and the nearest neighbour histogram within the training matrix found. BOP sets the three parameters through cross validation on the training data (Algorithm 12).
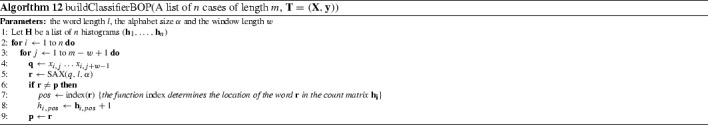


#### Symbolic aggregate approximation-vector space model (SAXVSM) (Senin and Malinchik 2013)

SAXVSM combines the SAX representation used in BOP with the vector space model commonly used in Information Retrieval. The key differences between BOP and SAXVSM is that SAXVSM forms term frequencies over classes rather than series and weights these by the inverse document frequency ($tf\cdot idf$). For SAXVSM, term frequency *tf* refers to the number of times a word appears in a class and document frequency *df* means the number of classes a word appears in. $tf\cdot idf$ is then defined as

$$\begin{aligned} tfidf(tf, df) = \left. {\left\{ \begin{array}{ll} \log {(1+tf)}\cdot \log \left( \frac{c}{ df }\right) &{} \quad \text {if } df > 0 \\ 0 &{} \quad otherwise \\ \end{array}\right. } \right. \end{aligned}$$

where *c* is the number of classes. SAXVSM is described formally in Algorithm 13. 
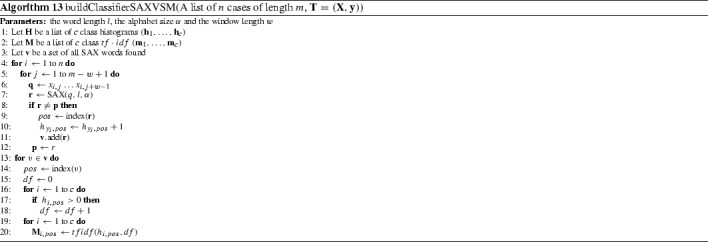
 Parameters *l*, $\alpha$ and *w* are set through cross validation on the training data. Predictions are made using a 1-NN classification based on the word frequency distribution of the new case and the $tf\cdot idf$ vectors of each class. The Cosine similarity measure is used.

####  Bag of SFA symbols (BOSS) (Schäfer 2015)

BOSS also uses windows to form words over series, but it has several major differences to BOP and SAXVSM. Primary amongst these is that BOSS uses a truncated Discrete Fourier Transform (DFT) instead of a PAA on each window. Another difference is that the truncated series is discretised through a technique called Multiple Coefficient Binning (MCB), rather than using fixed intervals. MCB finds the discretising break points as a preprocessing step by estimating the distribution of the Fourier coefficients. This is performed by segmenting the series, performing a DFT, then finding breakpoints for each coefficient so that each bin contains the same number of elements. BOSS then involves similar stages to BOP; it windows each series to form the term frequency through the application of DFT and discretisation by MCB. A bespoke distance function is used for nearest neighbour classification. This non symmetrical function only includes distances between frequencies of words that actually occur within the first histogram passed as an argument. BOSS also includes a parameter that determines whether the subseries are normalised or not.
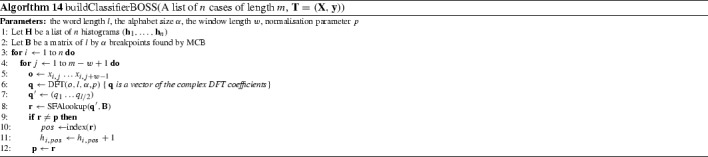


We evaluate the ensemble version of BOSS described in Schäfer (2015). During the parameter search of window sizes, the BOSS ensemble retains all classifiers with training accuracy within 92% of the best. New instances are classified by a majority vote (Algorithm 14).

**Fig. 7:**
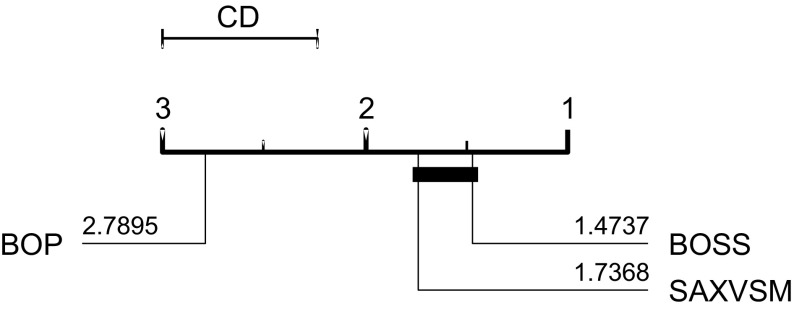
Average ranks of published results on 19 data sets or BOP, SAXVSM, BOSS

BOP and SAXVSM were evaluated on 20 and 19 UCR problems respectively. All algorithms used the standard single train/test split. BOSS presents results on an extended set of 58 data sets from a range of sources. On the 19 data sets they all have in common, BOP is significantly worse than BOSS and SAXVSM. There is no significant difference between BOSS and SAXVSM (see Fig. 7).

Our BOP results are not significantly different to the published ones. We were unable to reproduce as accurate results as published for SAXVSM and BOSS. On examination of the implementation for SAXVSM provided online and by correspondence with the author, it appears the parameters for the published results were obtained through optimisation on the test data. This obviously introduces bias, as can be seen from the results for Beef. An error of 3.3% was reported. This is far better than any other algorithm has achieved. Our results for BOSS are on average approximately 1% worse than those published, a significant difference. Correspondence with the author and examination of the code leads us to believe this is because of a versioning problem with the code that meant the normalisation parameter was set to minimize test data error rather than train error.

### Combinations of transformations

Ensembles have proved popular in recent TSC research and are highly competitive on general classification problems. TSF, TSBF and BOSS are ensembles based on the same core classifier. Other approaches, such as the EE and ST use different classifier components. All of these ensembles are constructed on the same representation. Two recently proposed algorithms that have published very promising results combine features and classifiers from different representations.

#### DTW features ($\hbox {DTW}_F$) (Kate 2016)

Kate (2016) proposes a feature generation scheme, $\hbox {DTW}_F$, that combines DTW distances to training cases and SAX histograms. $\hbox {DTW}_F$ combines whole series and dictionary based approaches into a single classifier. A training set with *n* cases is transformed into a set with *n* features, where feature $x_{ij}$ is the full window DTW distance between case *i* and case *j*. A further *n* features are then created. These are the optimal window DTW distance between cases. Finally, SAX word frequency histograms are created for each instance using the BOP algorithm. These $a^l$ features are concatenated with the 2*n* full and optimal window DTW features. The new dataset is trained with a support vector machine with a polynomial kernel with order either 1, 2 or 3, set through cross validation. DTW window size and SAX parameters are also set independently through cross validation with a 1-NN classifier. A more formal description is provided in Algorithm 15.
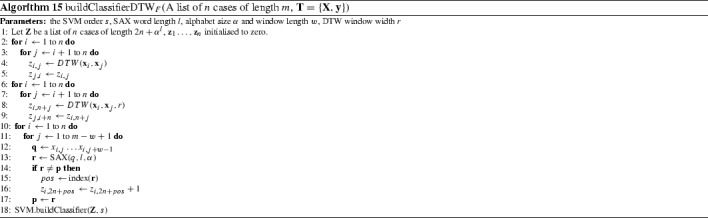


#### Collection of transformation ensembles (COTE) (Bagnall et al. 2015)

In terms of the taxonomy we describe in this paper, the only classifier we are aware of that explicitly ensembles over different representations is the collective of transformation ensembles (COTE). Bagnall et al. (2015) propose the meta ensemble COTE, a combination of classifiers in the time, autocorrelation, power spectrum and shapelet domains. The components of EE and ST are pooled with classifiers built on data transformed into autocorrelation (ACF) and power spectrum (PS) representations. EE uses the 11 classifiers described in Sect. 2.1.8. ACF and PS employ the same 8 classifiers used in conjunction with the shapelet transform. We use the classifier structure called flat-COTE in Bagnall et al. (2015). This involves pooling all 35 classifiers into a single ensemble with votes weighted by train set cross validation accuracy. There is however one difference: we use the Shapelet Transform described in Bostrom and Bagnall (2015) rather than the version in Hills et al. (2014).

$\hbox {DTW}_F$ is assessed on 47 UCR data, COTE on a set of 75 datasets that include the UCR data. We can reproduce the published results of $\hbox {DTW}_F$. We can reproduce the COTE results with the old version of ST, but the results with the new version of ST are significantly better.

### Time and space complexity

We reiterate that our primary concern is accuracy. We limit our consideration of timing experiments to the elastic measures (Sect. 5.1) because comparing all algorithms presents significant problems and potential biases. If we perform timings then we would need to ensure all our code was optimized, not just that for our own algorithms. We run all experiments on a shared cluster, so would need to mitigate for this. We cannot time on a single problem, because some data has characteristics that slow down certain algorithms. For example, LS has a condition where it restarts when the search degenerates. This is much more likely to happen on some problems (for some reason, ECG200 was the worst) than others. Nevertheless, there is massive variation in the time and memory these algorithms require, so consideration of the complexity of the algorithms is of interest. In particular, we found that little consideration has been given to space complexity in the literature, and we found this a serious problem for some algorithms. Table 1 summarises the time and space complexity of the 18 TSC algorithms considered in the study.

**Table 1:** Summary of the time and space complexity of the 18 TSC algorithms considered

	Train time	Train space	Parameters
WDTW	$O(n^2m^2)$	$O(m^2)$	
TWE	$O(n^2m^2)$	$O(m^2)$	
MSM	$O(n^2m^2)$	$O(m^2)$	
CID	$O(n^2m^2)$	$O(m^2)$	
$\hbox {DD}_{\textit{DTW}}$	$O(n^2m^2)$	$O(m^2)$	
$\hbox {DTD}_C$	$O(n^2m^2)$	$O(m^2)$	
EE	$O(n^2m^2)$	$O(m^2)$	
ST	$O(n^2m^4)$	*O*(*kn*)	*k*: number of shapelets
LS	$O(em^2n^2R^2)$	$O(m^2n^2R^2)$	*R*: shapelet scale, *e*: max nos iterations
FS	$O(nm^2)$	$O(nm^2)$	
TSF	$O(rmn\log n)$	*O*(*rm*)	*r*: number of trees
TSBF	$O(rmnw\log n)$	*O*(*rm*)	*w*: number of subseries
LPS	$O(rmnw\log n)$	*O*(*rm*)	
BOP	$O(nm(n-w))$	$O(n\alpha ^l)$	*w*: window length, *l*: word length
SAXVSM	$O(nm(n-w))$	$O(c\alpha ^l)$	$\alpha$: alphabet size
BOSS	$O(nm(n-w))$	$O(n\alpha ^l)$	
$\hbox {DTW}_F$	$O(n^2m^2)$	$O(n^2+n\alpha ^l)$	
COTE	$O(n^2m^4)$	$O(knm^2)$	

Train time includes the cross validated parameter search. Series length is *m*, number of series is *n* and number of classes is *c*

We can broadly categorise the algorithms by time and space complexity. The slowest algorithms are ST, EE and COTE. LS and TWE are also relatively slow on large problems. The fastest algorithms are FS, TSF, BOP and SAXVSM. The most memory intensive algorithms are $\hbox {DTW}_F$, BOSS, LS, ST and COTE. The memory requirements for the shapelet algorithms can be reduced by the simple expedient of finding fewer shapelets for ST.

### Summary

We have grouped the algorithms into those that use the whole series, intervals, shapelets or repeating pattern counts (see Table 2).

**Table 2:** A summary of algorithm taxonomy by data feature characteristics: weighted DTW (WDTW) (Jeong et al. 2011); time warp edit (TWE) (Marteau 2009); move–split–merge (MSM) (Stefan et al. 2013); complexity invariant distance (CID) (Batista et al. 2014); derivative DTW ($\hbox {DD}_{\textit{DTW}}$) (Górecki and Łuczak 2013); derivative transform distance ($\hbox {DTD}_C$) (Górecki and Łuczak 2014); elastic ensemble (EE) (Lines and Bagnall 2015); time series forest (TSF) (Deng et al. 2013); time series bag of features (TSBF) (Baydogan et al. 2013); learned pattern similarity (LPS) (Baydogan and Runger 2016); fast shapelets (FS) (Rakthanmanon and Keogh 2013); shapelet transform (ST) (Hills et al. 2014); bag of patterns (BOP) (Lin et al. 2012); SAX vector space model (SAXVSM) (Senin and Malinchik 2013); bag of SFA symbols (BOSS) (Schäfer 2015); DTW features (Kate 2016); collective of transformation-based ensembles (COTE) (Bagnall et al. 2015)

Whole series	Intervals	Shapelets	Dictionary
WDTW	TSF	FS	BOP
TWE	TSBF	ST	SAXVSM
MSM	LPS	LS	BOSS
CID		COTE	$\hbox {DTW}_F$
$$\hbox {DD}_{\textit{DTW}}$$			
$$\hbox {DTD}_C$$			
EE			
COTE			

The point of any grouping is to aid understanding of the techniques. There are many alternative classifications we could employ. We could label based on the type of classifier (most simply, nearest neighbour or not). We could split the whole series measures into those that use derivatives or not. We could distinguish between those that use the autocorrelation function or not. Table 3 gives the break down of algorithms versus these criteria.

There are many other approaches that have been proposed that we have not included due to time constraints and failure to meet our inclusion criteria. Two worthy of mention are Silva et al.’s recurrence plot compression distance (RPCD) (Silva et al. 2013) and Fulcher and Jones’s feature-based linear classifier (FBL) (Fulcher and Jones 2014). RPCD involves transforming each series into a 2 dimensional recurrence plot then measuring similarity based on the size of the MPEG1 encoding of the concatenation of the resulting images. We were unable to find a working Java based MPEG1 encoder, and the technique seems not to work with the MPEG4 encoders we tried. FBL involves generating a huge number of possible features which are filtered with a forward selection mechanism for a linear classifier. The technique utilises built in matlab functions to generate thousands of features. Unfortunately these functions are not readily available in Java, and we considered it infeasible to attempt such as colossal task. It is further worth noting that COTE produces significantly better results than both RPCD and FBL (Bagnall et al. 2015).

**Table 3:** A summary of algorithms and the component approaches underlying them

	NN	time	deriv	shape	int	dict	auto	ens
WDTW	x	x						
TWE	x	x						
MSM	x	x						
CID	x	x	x					
$\hbox {DD}_{\textit{DTW}}$	x	x	x					
$\hbox {DTD}_C$	x	x	x					
ST				x				x
LS				x				
FS				x				
TSF					x			x
TSBF					x			x
LPS	x		x		x		x	
BOP	x					x		
SAXVSM	x					x		
BOSS	x					x	x	x
$\hbox {DTW}_F$	x	x				x		x
EE	x	x	x					x
COTE	x	x	x	x			x	x

Approaches are nearest neighbour classification (NN), time domain distance function (time), derivative based distance function (deriv), shapelet based (shape), interval based (int), dictionary based (dict), auto-correlation based (auto) and ensemble (ens)

## Data and experimental design

**Fig. 8:**
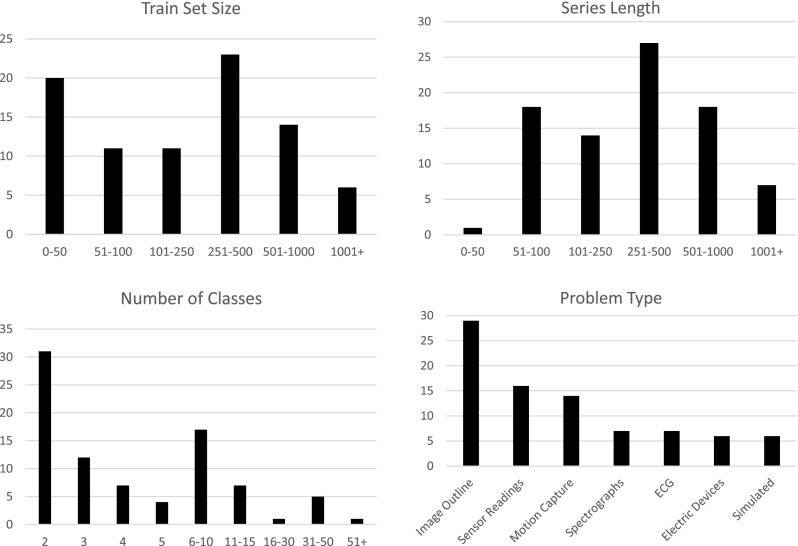
Summary information for the 85 datasets in the archive

The 85 datasets are described in detail on the website (Bagnall et al., http://timeseriesclassification.com). The collection is varied in terms of data characteristics: the length of the series ranges from 24 (ItalyPowerDemand) to 2709 (HandOutlines); train set sizes vary from 16 to 8926; and the number of classes is between 2 and 60. Figure 8 shows more detailed histograms. There are a large number of datasets with small train set sizes (twenty have 50 or fewer train instances). Whilst this is historically valid (labelled data is often expensive to collect), data is becoming cheaper and ubiquitous, so we believe that larger datasets should be better represented in the future.

The data are from a wide range of domains, with an over representation of image outline classification problems. We are introducing four new food spectra data sets: Ham; Meat; Strawberry; and Wine. These were all created by the Institute of Food Research, part of the Norwich Research Park, as were the three spectra data already in the UCR repository (Beef, Coffee and OliveOil). Figure 8 gives the breakdown of number of problems per category.

We run the same 100 resample folds on each problem for every classifier. The first fold is always the original train test split. The other resamples are stratified to retain class distribution in the original train/trest splits. These resample datasets can be exactly reproduced using the method InstanceTools.resampleTrainAndTestInstances with seeds 0–99.

Each classifier must be evaluated 8500 times (100 resamples on 85 datasets). Model selection is repeated on every training set fold. We used the parameter values searched in the relevant publication as closely as possible. The parameter values we search are listed in Table 4. We allow each classifier a maximum 100 parameter values, each of which we assess through a cross validation on the training data. The number of cross validation folds is dependent on the algorithm. This is because the overhead of the internal cross validation differs. For the distance based measures it is as fast to do a leave-one-out cross validation as any other. For others we need a new model for each set of parameter values. This means we need to construct 850,000 models for each classifier. When we include repetitions caused by bugs, we estimate we have conducted over 30 million distinct experiments over 6 months.

The datasets vary greatly in size. The eight largest and invariably the slowest are: ElectricalDevices; FordA; FordB; HandOutlines; NonInvasiveFetalECGThorax1; NonInvasiveFetalECGThorax2; StarlightCurves; and UWaveGestureLibraryAll. We have had to sub-sample these data sets for the model selection stages for the slower or memory intensive algorithms such as ST, LS and BOSS. Full details of the sampling performed are in the code documentation.

**Table 4:** Parameter settings and ranges for TSC algorithms

	Parameters	CV Folds
WDTW	$g \in \{0,0.01,\ldots ,1\}$	LOOCV
TWE	$\nu \in \{0.00001, 0.0001, 0.001 ,0.01, 0.1, 1\}$ and $\lambda \in \{0, 0.25, 0.5, 0.75, 1.0\}$	LOOCV
MSM	$c \in \{0.01, 0.1, 1, 10, 100\}$	LOOCV
CID	$r \in \{0.01, 0.1, 1, 10, 100\}$	LOOCV
$\hbox {DD}_{\textit{DTW}}$	$a \in \{0,0.01,\ldots ,1\}$	LOOCV
$\hbox {DTD}_C$	$a \in \{0,0.1,\ldots ,1\}$, $b \in \{0,0.1,\ldots ,1\}$	LOOCV
ST	min=3, max=m-1 $$k=10n $$	0
LS	$\lambda \in \{0.01,0.1\}$, $L \in \{0.1,0.2\}$, $R \in \{2,3\}$	3
FS	$r=10$, $k=10$, $l= 16$ and $\alpha = 4$	0
TSF	$r=500$	0
TSBF	$z \in \{0.1,0.25,0.5,0.75\}$, $a=5$, $b=10$	LOOCV
LPS	$w=20$, $d \in {2,4,6}$,	LOOCV
BOP	$\alpha \in {2, 4, 6, 8}$, w from 10 to 36% of *m*, $l \in {2^i | i=1 to \log (w/2)}$	LOOCV
SAXVSM	$\alpha \in {2, 4, 6, 8}$, w from 10 to 36% of *m*, $l \in {2, 4, 6, 8}$	LOOCV
BOSS	$\alpha =4$, *w* from 10 to *m*, with $\min (200,\sqrt{(}m))$, $l \in {8, 10, 12, 14, 16}$	LOOCV
$\hbox {DTW}_F$	DTW paras 0 to 0.99, SAX pars as BOP, SVM kernel degree $\{1,2,3\}$	10
EE	Constituent classifier parameters only	0
COTE	Constituent classifier parameters only	0

The notation is overloaded in order to maintain consistency with authors’ original parameter names

We follow the basic methodology described in Demšar (2006) when testing for significant difference between classifiers. For any single problem we can compare differences between two or more classifiers over the 100 resamples using standard parametric tests (t-test for two classifiers, ANOVA for multiple classifiers) or non parametric test (binomial test or the Wilcoxon sign rank test for two classifiers, Friedman test for multiple classifiers). However, the fact we are resampling data means the observations are not independent and we should be careful interpreting too much into the results for a single problem. The real benefit of resampling is to reduce the risk of bias introduced through overfitting on a single sample. Our main focus of interest is relative performance over multiple data sets. Hence, we average accuracies over all 100 resamples, then compare classifiers by ranks using the Friedman test, which detects any overall difference in ranks. Following recommendations in Benavoli et al. (2016) and García and Herrera (2008), we then compare all classifiers with pairwise Wilcoxon signed rank tests, and form cliques using the Holm correction, which adjusts family-wise error less conservatively than a Bonferonni adjustment. We present the average ranks in a critical difference diagram (for example, see Figs. 3, 5, 7). These diagrams show the average ranks of the classifiers in order, and groupings, or cliques, represented by a solid bar. A clique represents a group of classifiers within which there is no significant pairwise difference.

## Overall results

Due to space constraints, we present an analysis of our results rather than presenting the full data. All of our results and spreadsheets to derive the graphs are available from (Bagnall et al., http://timeseriesclassification.com). All accuracies presented are absolute and not relative to each other. i.e., if we claim algorithm A is 10% better than algorithm B, we mean the average accuracy is 0.1 higher for algorithm A than B, not that it is 10% larger than B.

### Benchmark classifiers

We believe that newly proposed algorithms should add some value in terms of accuracy or efficiency over sensible standard approaches which are generally much simpler and better understood. The most obvious starting point for any classification problem is to use a standard classifier that treats each series a vector (i.e. make no explicit use of any autocorellation structure). Three characteristics that make TSC problems hard are having few cases, long series (large number of attributes) and highly correlated or redundant features. These are problems that are well studied in machine learning and classifiers have been designed to compensate for them. TSC characteristics that will confound traditional classifiers include discriminatory features in the autocorrelation function, phase independence within a class and embedded discriminatory subseries. However, not all problems will have these characteristics, and benchmarking against standard classifiers may give insights into the problem characteristics. We have experimented with WEKA versions of C4.5 (C45), naive Bayes (NB), logistic regression (logistic), support vector machines with linear (SVML) and quadratic kernel (SVMQ), multilayer perceptron (MLP), random forest (with 500 trees) (RandF) and rotation forest (with 50 trees) (RotF). In TSC specific research, the starting point with most investigations is 1-NN with Euclidean distance (ED). This basic classifier is a very low benchmark for comparison and is easily beaten with other standard classifiers. A more useful benchmark is 1-NN dynamic time warping with a warping window set through cross validation (DTW) (Ratanamahatana and Keogh 2005).

**Fig. 9:**
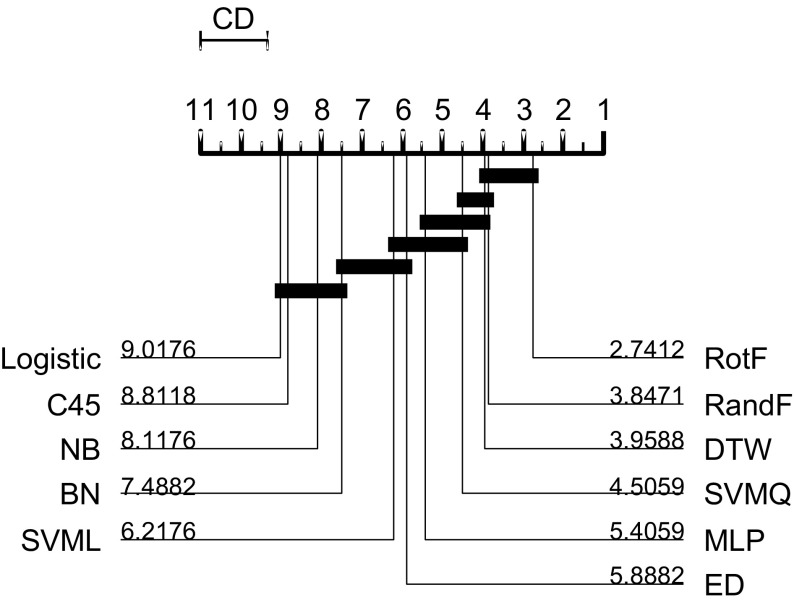
Critical difference diagram for 11 potential benchmark classifiers

Figure 9 shows the average ranks of the classifiers in a critical difference diagram. RotF, RandF and DTW form a clique of classifiers better than the others. Based on these results, we select RotF and DTW as our two benchmarks classifiers. Head to head, RotF has significantly better accuracy on 43 problems, DTW on 33, and no difference on 9 data sets.

### Comparison against benchmark classifiers

Table 5 shows the summary of the pairwise results of the 19 classifiers against DTW and RotF. The results largely confirm our prior beliefs. Of those significantly better than DTW, there is a group that are more accurate by some margin: COTE, EE, ST and BOSS are 3–8% more accurate than DTW on average. There is a second group that offer 1–3% improvement in accuracy (LPS, MSM, TSF, TSBF and $\hbox {DTW}_F$) and the final cluster offers a very small, but significant, improvement over DTW (WDTW, LS, $\hbox {DTD}_C$ and $\hbox {CID}_{\textit{DTW}}$). Rotation forest is actually harder to beat. The third group that offer small but significant improvement over DTW fail to outperform RotF. Nine classifiers are significantly better than both benchmarks: COTE; ST; BOSS; EE; $\hbox {DTW}_F$; TSF; TSBF; LPS; and MSM. BOP, SAXVSM and FS are all significantly worse than both the benchmarks.

**Table 5:** A summary of algorithm performance based on significant difference to DTW and Rotation Forest

Comparison to DTW			Comparison to RotF		
Classifier	Prop better (%)	Mean difference (%)	Classifier	Prop better (%)	Mean difference (%)
Significantly better than DTW			Significantly better than RotF		
COTE	96.47	8.12	COTE	84.71	8.14
EE	95.29	3.51	ST	75.29	6.15
ST	80.00	6.13	TSF	63.53	1.93
BOSS	78.82	5.67	BOSS	62.35	5.70
$\hbox {DTW}_F$	75.29	2.87	LPS	60.00	1.86
TSF	68.24	1.91	EE	58.82	3.54
TSBF	65.88	2.19	$\hbox {DTW}_F$	58.82	2.89
MSM	62.35	1.89	MSM	57.65	1.91
LPS	61.18	1.83	TSBF	56.47	2.22
WDTW	60.00	0.20	Not significantly different to RotF		
LS	58.82	0.56	LS	61.18	0.58
$\hbox {DTD}_C$	52.94	0.79	$\hbox {CID}_{\textit{DTW}}$	48.24	0.56
$\hbox {CID}_{\textit{DTW}}$	50.59	0.54	$\hbox {DTD}_C$	47.06	0.82
Not significantly different to DTW			$\hbox {DD}_{\textit{DTW}}$	45.88	0.44
$\hbox {DD}_{\textit{DTW}}$	56.47	0.42	TWE	45.88	0.40
RotF	56.47	$-$0.02	WDTW	44.71	0.22
TWE	49.41	0.37	DTW	43.53	0.02
Significantly worse than DTW			Significantly worse than RotF		
SAXVSM	41.18	$-$3.29	BOP	34.12	$-$3.03
BOP	37.65	$-$3.05	SAXVSM	31.76	$-$3.26
FS	30.59	$-$7.40	FS	22.35	$-$7.38

The column prop better gives the proportion of problems where the classifier has a significantly higher mean accuracy over 100 resamples than the benchmark. The column mean gives the mean difference in mean accuracy over all 85 problems. Thus, for example, COTE is on average 8.12% more accurate than DTW over the 85 problems

### Comparison of TSC algorithms

**Fig. 10:**
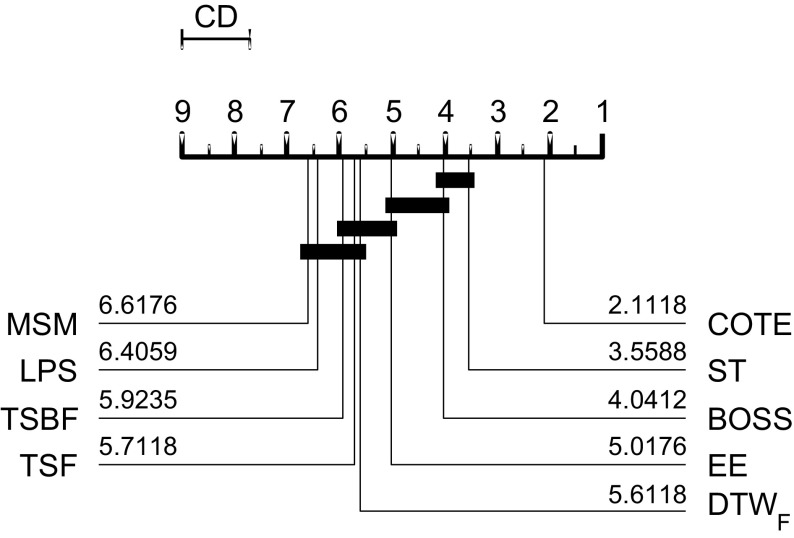
Critical difference diagram for the nine classifiers significantly better than both benchmark classifiers

Table 6 gives the mean accuracy and standard deviation (over 85 problems) of the nine classifiers that beat the benchmarks. The rank data is given in Fig. 10.

**Table 6:** Average accuracy of the best nine classifiers over 85 problems

Datasets	COTE	ST	BOSS	EE	$\hbox {DTW}_F$	TSF	TSBF	LPS	MSM
Adiac	**0**.**81**	0.768	0.749	0.665	0.605	0.707	0.727	0.765	0.636
ArrowHead	**0**.**877**	0.851	0.875	0.86	0.776	0.789	0.801	0.806	0.815
Beef	**0**.**764**	0.736	0.615	0.532	0.546	0.648	0.554	0.52	0.474
BeetleFly	0.921	0.875	**0**.**949**	0.823	0.853	0.842	0.799	0.893	0.794
BirdChicken	0.941	0.927	**0**.**984**	0.848	0.865	0.839	0.902	0.854	0.866
Car	0.899	**0**.**902**	0.855	0.799	0.851	0.758	0.795	0.836	0.841
CBF	0.998	0.986	**0**.**998**	0.993	0.979	0.958	0.977	0.984	0.972
ChlorineConcentration	**0**.**736**	0.682	0.66	0.659	0.658	0.719	0.683	0.642	0.626
CinCECGtorso	**0**.**983**	0.918	0.9	0.946	0.714	0.974	0.716	0.743	0.935
Coffee	**1**	0.995	0.989	0.989	0.973	0.989	0.982	0.95	0.945
Computers	0.77	0.785	**0**.**802**	0.732	0.659	0.768	0.765	0.726	0.713
CricketX	**0**.**814**	0.777	0.764	0.801	0.769	0.691	0.731	0.696	0.778
CricketY	**0**.**815**	0.762	0.749	0.794	0.756	0.688	0.728	0.706	0.76
CricketZ	**0**.**827**	0.798	0.776	0.804	0.785	0.707	0.738	0.714	0.779
DiatomSizeReduction	0.925	0.911	0.939	**0**.**946**	0.942	0.941	0.89	0.915	0.939
DistalPhalanxOAG	0.805	**0**.**829**	0.815	0.768	0.796	0.809	0.816	0.767	0.756
DistalPhalanxOC	**0**.**821**	0.819	0.814	0.768	0.76	0.813	0.812	0.742	0.754
DistalPhalanxTW	**0**.**693**	0.69	0.673	0.654	0.658	0.686	0.69	0.618	0.618
Earthquakes	0.747	0.737	0.746	0.735	**0**.**747**	0.747	0.747	0.668	0.695
ECG200	0.873	0.84	**0**.**89**	0.881	0.819	0.868	0.847	0.807	0.877
ECG5000	**0**.**946**	0.943	0.94	0.939	0.94	0.944	0.938	0.917	0.93
ECGFiveDays	**0**.**986**	0.955	0.983	0.847	0.907	0.922	0.849	0.84	0.879
ElectricDevices	0.883	**0**.**895**	0.8	0.831	0.874	0.804	0.808	0.853	0.825
FaceAll	**0**.**99**	0.968	0.974	0.976	0.963	0.949	0.942	0.962	0.986
FaceFour	0.85	0.794	**0**.**996**	0.879	0.909	0.891	0.862	0.889	0.92
FacesUCR	0.967	0.909	0.951	0.948	0.889	0.897	0.849	0.91	**0**.**97**
Fiftywords	0.801	0.713	0.702	**0**.**821**	0.748	0.728	0.744	0.776	0.817
Fish	0.962	**0**.**974**	0.969	0.913	0.931	0.807	0.913	0.912	0.897
FordA	0.955	**0**.**965**	0.92	0.751	0.884	0.816	0.831	0.869	0.725
FordB	**0**.**929**	0.915	0.911	0.757	0.843	0.79	0.751	0.852	0.73
GunPoint	0.992	**0**.**999**	0.994	0.974	0.964	0.962	0.965	0.972	0.948
Ham	0.805	0.808	**0**.**836**	0.763	0.795	0.795	0.711	0.685	0.745
HandOutlines	0.894	**0**.**924**	0.903	0.88	0.915	0.909	0.879	0.868	0.864
Haptics	**0**.**517**	0.512	0.459	0.451	0.464	0.467	0.463	0.415	0.444
Herring	0.632	**0**.**653**	0.605	0.566	0.609	0.606	0.59	0.549	0.559
InlineSkate	**0**.**526**	0.393	0.503	0.476	0.382	0.379	0.377	0.449	0.455
InsectWingbeatSound	**0**.**639**	0.617	0.51	0.581	0.602	0.613	0.616	0.519	0.57
ItalyPowerDemand	**0**.**97**	0.953	0.866	0.951	0.948	0.958	0.926	0.914	0.936
LargeKitchenAppliances	0.9	**0**.**933**	0.837	0.816	0.823	0.644	0.551	0.68	0.749
Lightning2	0.785	0.659	0.81	**0**.**835**	0.71	0.757	0.76	0.757	0.792
Lightning7	**0**.**799**	0.724	0.666	0.763	0.671	0.723	0.68	0.631	0.713
Mallat	**0**.**974**	0.972	0.949	0.961	0.929	0.937	0.951	0.908	0.918
Meat	0.981	0.966	0.98	0.978	**0**.**983**	0.978	0.983	0.968	0.977
MedicalImages	**0**.**785**	0.691	0.715	0.761	0.701	0.757	0.701	0.71	0.757
MiddlePhalanxOAG	0.801	**0**.**815**	0.808	0.782	0.798	0.794	0.8	0.77	0.751
MiddlePhalanxOC	**0**.**722**	0.694	0.666	0.609	0.581	0.676	0.673	0.597	0.56
MiddlePhalanxTW	**0**.**587**	0.579	0.537	0.525	0.519	0.577	0.568	0.503	0.499
MoteStrain	0.902	0.882	0.846	0.875	0.891	0.874	0.886	**0**.**917**	0.88
NonInvFetalECGThorax1	0.929	**0**.**947**	0.841	0.849	0.877	0.88	0.842	0.807	0.818
NonInvFetalECGThorax2	0.946	**0**.**954**	0.904	0.914	0.898	0.914	0.862	0.826	0.894
OliveOil	**0**.**901**	0.881	0.87	0.879	0.864	0.883	0.864	0.892	0.872
OSULeaf	0.949	0.934	**0**.**967**	0.812	0.809	0.637	0.678	0.763	0.787
PhalangesOutlinesCorrect	0.783	0.794	0.821	0.78	0.793	0.804	**0**.**825**	0.79	0.76
Phoneme	**0**.**362**	0.329	0.256	0.299	0.22	0.211	0.278	0.245	0.275
Plane	**1**	1	0.998	**1**	0.996	0.994	0.993	1	0.999
ProximalPhalanxOAG	0.871	**0**.**881**	0.867	0.839	0.829	0.847	0.861	0.851	0.806
ProximalPhalanxOC	**0**.**848**	0.841	0.819	0.805	0.824	0.846	0.842	0.8	0.769
ProximalPhalanxTW	**0**.**815**	0.803	0.773	0.759	0.774	0.808	0.798	0.722	0.729
RefrigerationDevices	0.742	0.761	**0**.**785**	0.676	0.656	0.615	0.638	0.675	0.704
ScreenType	0.651	**0**.**676**	0.586	0.554	0.499	0.573	0.538	0.506	0.493
ShapeletSim	0.964	0.934	**1**	0.827	0.888	0.51	0.913	0.874	0.85
ShapesAll	**0**.**911**	0.854	0.909	0.886	0.796	0.8	0.853	0.885	0.875
SmallKitchenAppliances	0.788	0.802	0.75	0.703	0.753	**0**.**813**	0.674	0.724	0.717
SonyAIBORobotSurface1	**0**.**899**	0.888	0.897	0.794	0.884	0.845	0.839	0.842	0.764
SonyAIBORobotSurface2	**0**.**96**	0.924	0.888	0.87	0.859	0.856	0.825	0.851	0.877
StarlightCurves	**0**.**98**	0.977	0.978	0.941	0.96	0.969	0.978	0.968	0.882
Strawberry	0.963	0.968	**0**.**97**	0.959	0.97	0.963	0.968	0.963	0.958
SwedishLeaf	**0**.**967**	0.939	0.918	0.916	0.885	0.892	0.908	0.926	0.887
Symbols	0.953	0.862	**0**.**961**	0.957	0.93	0.888	0.944	0.96	0.952
SyntheticControl	**0**.**999**	0.987	0.968	0.994	0.986	0.99	0.987	0.972	0.982
ToeSegmentation1	0.934	**0**.**954**	0.929	0.788	0.922	0.661	0.858	0.841	0.821
ToeSegmentation2	0.951	0.947	**0**.**96**	0.907	0.904	0.782	0.886	0.926	0.895
Trace	**1**	1	1	0.996	0.997	0.998	0.981	0.966	0.956
TwoLeadECG	0.983	0.984	**0**.**985**	0.958	0.958	0.842	0.91	0.928	0.941
TwoPatterns	1	0.952	0.991	**1**	1	0.991	0.974	0.967	0.999
UWaveGestureLibraryX	0.831	0.806	0.753	0.805	0.806	0.806	**0**.**834**	0.819	0.775
UWaveGestureLibraryY	**0**.**766**	0.737	0.661	0.731	0.717	0.727	0.746	0.753	0.69
UWaveGestureLibraryZ	0.76	0.747	0.695	0.726	0.736	0.741	**0**.**776**	0.766	0.701
UWaveGestureLibraryAll	**0**.**965**	0.942	0.944	0.968	0.963	0.962	0.944	0.968	0.96
Wafer	0.999	**1**	0.999	0.997	0.996	0.997	0.996	0.995	0.996
Wine	0.904	**0**.**926**	0.912	0.887	0.892	0.881	0.879	0.884	0.884
WordSynonyms	0.748	0.582	0.659	**0**.**778**	0.674	0.643	0.669	0.728	0.773
Worms	0.725	0.719	**0**.**735**	0.644	0.673	0.628	0.668	0.642	0.616
WormsTwoClass	0.785	0.779	**0**.**81**	0.717	0.73	0.685	0.755	0.743	0.712
Yoga	0.898	0.823	**0**.**91**	0.885	0.863	0.867	0.835	0.874	0.888
Average rank	2.11	3.56	4.04	5.02	5.61	5.71	5.92	6.40	6.62
Wins	36.5	17	17	6.5	2	1	3	1	1

The best algorithm of these nine is in bold. Some of the problem names are abbreviated and all of the results are rounded to 3 decimal places to save space. Apparent discrepancies such as the fact ST has accuracy of 1 on plane but is not registered as one of the best are caused by rounding (ST average accuracy is 0.99961). Full results, including the accuracy for each fold for every algorithm, are available on the website

The most obvious conclusion from this graph is that COTE is significantly better than the others. COTE performs best on 36 problems. EE and ST are components of COTE, hence this result demonstrates the benefits of combining classifiers on alternative feature spaces. We believe the success of COTE is based on the fact that the wide range of problem types we consider means that no one representation will dominate. Table 7 shows the difference of the main algorithms from COTE and also the standard deviation in ranks of the top nine algorithms and the two benchmarks.

**Table 7:** Average deviation in accuracy from COTE, standard deviation in ranks and maximum rank over all 85 data sets

Algorithm	Difference to COTE	Rank Standard Deviation	Max Rank
COTE	0	3.81	22
ST	$-$2.00%	7.89	33
BOSS	$-$2.45%	6.93	35
EE	$-$4.61%	5.79	26
$\hbox {DTW}_F$	$-$5.25%	6.57	30
TSF	$-$6.21%	7.27	32
TSBF	$-$5.93%	7.37	33
LPS	$-$6.29%	9.11	35
MSM	$-$6.23%	8.79	37
RotF	$-$8.14%	9.84	37
DTW	$-$8.12%	8.07	36

COTE, EE and $\hbox {DTW}_F$ have relatively low deviation in ranks. COTE and $\hbox {DTW}_F$ are the only algorithms that span different representations and EE combines whole series measures on both time domain and first order differences. Conversely, ST varies in rank considerably, even though it is an ensemble and ranked second overall. Shapelets work well on some problems but poorly on others where the approach is inappropriate. The second point Table 7 highlights is that even though COTE is on average better, this does not necessarily help when given a specific problem. The fact that COTE is significantly better than the others overall does not mean it will always be a good choice. On FaceFour, for example, it is ranked 22 of the 37 algorithms we evaluate and is beaten by algorithms such as a Bayesian Network. Similarly, BOSS is ranked 35th on ItalyPowerDemand. There are two obvious questions that would aid practitioners of TSC. Firstly, is there a problem characteristic that would allow us to make an informed choice as to the best classifier? Secondly, can we choose the algorithm based on performance on the train data? The latter question can be partially addressed through mechanisms such as Texas sharpshooter plots (Batista et al. 2014). Where possible, we address this in Sect. 5. However, for some algorithms it is just not feasible to get an unbiased train set accuracy estimate. The computation required to perform holdout shapelet transforms for ST (and hence COTE) is unfeasible. Similarly, algorithms that perform extensive model selection would require another layer of cross validation. The first question we address in the following section.

**Table 8:** Best performing algorithms split by problem type

Problem	COTE (%)	Dictionary (%)	Elastic (%)	Interval (%)	Shapelet (%)	Vector (%)	#
Image outline	20.69	17.24	**24.14**	0.00	17.24	20.69	29
Sensor readings	**38.89**	0.00	16.67	5.56	22.22	16.67	18
Motion capture	**35.71**	21.43	14.29	14.29	14.29	0.00	14
Spectrographs	0.00	0.00	0.00	0.00	0.00	**100.00**	7
Electric devices	0.00	33.33	0.00	16.67	**50.00**	0.00	6
ECG	33.33	16.67	0.00	0.00	**50.00**	0.00	6
Simulated	**40.00**	20.00	20.00	0.00	20.00	0.00	5

Each entry is the percentage of problems of that type a member of a class of algorithm is most accurate for

Bold values indicate the largest value on the row (i.e. the best performing algorithm for each problem type)

### What does the problem type tell us about the best algorithm type?

Table 8 shows the performance of algorithms against problem type. The data is meant to give an indication as to which family of approaches may be best for each problem type. The sample sizes are small, so we must be careful drawing too many conclusions. However, this table does indicate how evaluation can give insights into problem domains. Perhaps the most important illustration of understanding the problem domain is the fact that vector classifiers are best on all of the spectrographic data sets. Spectral data are generally unlikely to have much phase shifting, so this makes sense in terms of the application. Shapelets are best on 3 out of 6 of the ElectricDevice problems and 3 out of 6 ECG datasets, but only 26% of problems overall. This makes sense in terms of the applications, because the profile of electricity usage and ECG irregularity will be a subseries of the whole and largely phase independent. COTE wins a relatively high proportion of sensor and motion data sets. This implies this type of problem is likely to have descriminatory features in multiple domains and no one representation is likely to be sufficient.

### What do the characteristics of the problem tell us about the best algorithm type?

It would be highly desirable if characteristics of the problem type such as train set size, series length and number of classes could provide guidance on the best algorithm for a specific problem. Accuracy across different problems is not comparable, so we look for patterns of relative performance by restricting our attention to the average ranks for different treatment levels of series length, number of classes and train set size. This exploratory analysis is far from definitive, but it may give insights into algorithm selection and indicate areas where algorithms could be improved. We only look at the individual effect of each characteristic because we do not have a large enough sample size to usefully assess interactions.

**Fig. 11:**
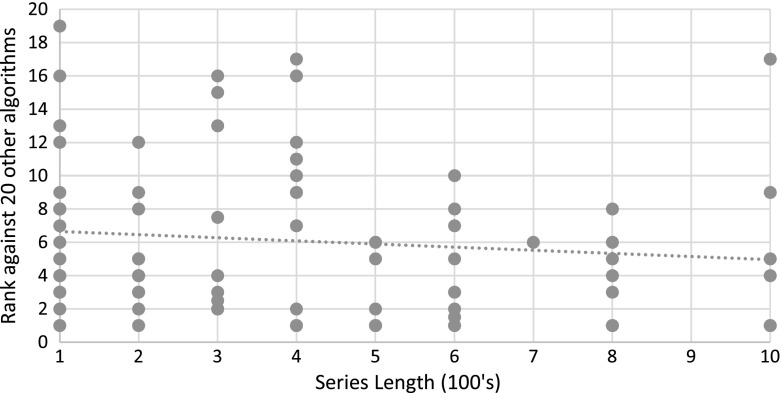
The rank of BOSS against series length, grouped into sets with lengths 1–100, 101–200, etc. The last group contains all series of length 1000 or more. The *dotted line* is the linear regression fit

Series length is the least informative data characteristic. For the majority of problems, the length of the series is an artifact of the preprocessing. For example, consider the StarLightCurves dataset. The series are of length 1024, but most are created from just a few dozen data points extrapolated to 1024 data points. If one down samples these objects to 512, 256, or even 128, it does not have a statistically significant effect on accuracy. Similarly, one of the longest series is HandOutlines. This data set was created from a set of outlines of radiographs, each of which was of different lengths. It was decided to up sample each outline to the length of the longest, but no extra information is added. The only really noticeable effect of series length is that the benchmark RotationForest gets relatively worse as series length increases whereas the dictionary methods get better, although this effect is small. Figure 11 shows the rank of BOSS for the 85 datasets grouped by series length. There is a very slight downward trend, but much higher variation within each group.

There is wide variation in the way TSC algorithms deal with multi-class problems, so we would expect some variation in relative performance as the number of classes varies. We group datasets into those with 2 classes (31 problems), 3 classes (12 problems), 4–5 classes (11 problems), 6–7 classes (11 problems), 8–12 classes (10 problems) and $13+$ classes (10 problems). For each algorithm we calculate the average rank over each treatment level. An analysis of variance indicates there is a significant variation difference in ranks between the class groupings. Table 9 shows the average rank of nine algorithms factored by number of classes. MSM, EE and COTE all improve relatively as the number of classes increases, whereas $\hbox {DTW}_F$, BOSS and ST all get worse. Could this be caused by characteristics of the algorithms? It is hard to say, but for ST this is likely to be true. The latest version of ST balances the number of shapelets for each class to avoid the problem of good shapelets from a small number of classes swamping the transform, but this is a fairly crude control mechanism. It is still likely that there are too many shapelets from the easy to classify classes excluding potentially useful ones from the hard to classify classes.

**Table 9:** Algorithm ranks split by number of classes

#Classes	MSM	LPS	TSBF	TSF	$\hbox {DTW}_F$	EE	BOSS	ST	COTE
2	11.94	10.74	9.76	9.53	8.31	8.97	4.26	4.55	3.16
3	12.92	10.42	8.42	8.83	8.50	7.67	3.67	4.00	2.92
4–5	10.82	12.09	11.64	7.00	8.45	7.45	6.41	7.05	3.91
6–7	10.91	11.27	10.09	10.45	9.73	5.77	7.36	6.05	2.05
8–12	8.70	10.10	8.30	9.10	7.90	4.00	11.70	6.00	1.50
13+	6.00	7.19	9.63	11.31	11.94	4.00	7.06	6.63	1.81

Each data represents the average rank of that algorithm over all problems with the range of classes shown in the first column

Given that many of the algorithms employ nearest neighbour classification, we would expect train set size to also have an influence on relative performance. Table 10 presents the average ranks of the top algorithms over four groups of train set size. We observe the interval based methods TSBF and TSF all improve whereas the elastic time domain method MSM gets worse. The MSM performance is broadly observable in the other elastic techniques such as TWE and WDTW. We believe this is caused by the fact they all use a single neighbour to classify, thus ignoring the extra information available in the larger datasets. This idea is supported by the fact the drop in performance is less observable in EE, which ensembles numerous 1-NN classifiers. A switch to *k* nearest neighbours may improve the relative performance of all the elastic classifiers on larger train sets.

**Table 10:** Algorithm ranks split by train set sizes

#Train cases	MSM	LPS	TSBF	TSF	$\hbox {DTW}_F$	EE	BOSS	ST	COTE
${<}$100 (28)	10.89	10.86	11.23	11.13	9.52	7.79	5.64	6.04	3.39
100–399 (28)	8.96	11.61	11.18	10.36	9.57	5.98	5.66	5.75	2.09
400–799 (15)	12.40	10.73	6.73	6.93	8.80	7.53	5.60	4.33	2.53
${>}$799 (14)	12.00	7.71	7.29	6.21	6.07	7.00	7.93	4.29	2.86

Each data represents the average rank of that algorithm over all problems with the range of train set size shown in the first column. The number of datasets in each category is in brackets

## Within algorithm type comparison

One of our central arguments is that different problems require different representations, and the reason COTE is so accurate is that it combines classifiers constructed on different transformations. If COTE is computationally infeasible, we think the choice of the correct family of algorithms is at least as important as the choice of which algorithm given a particular representation. To help understand the variation of techniques by grouping, we look at the relative performance of algorithms without COTE and $\hbox {DTW}_F$ (which both use more than one representation).

Table 11 gives the relative performance of algorithms of different classes grouped by the optimal for each problem type. The table is meant to highlight the importance of utilising the correct technique and highlights the similarity of the approaches. Another reason for its inclusion is to highlight that there are some large variations in accuracy. 5 to 10% differences are commonplace, particularly between classifier types.

A smaller difference indicates a similarity of performance between the two groups. So, for example, the best of the standard vector based classifiers are on average 15% less accurate on problems where the dictionary technique is the best and 10% worse where shapelets wins. The best shapelet approach is only 2% less accurate when interval methods are optimal. This can be explained by the fact that intervals can also be shapelets. However, the opposite is not true. Interval methods are 5.54% worse on problems best approached with shapelets. This highlights that the potential phase independence of shapelets is crucial. Shapelets do relatively badly when whole series elastic measures do best, and vice versa. Perhaps more surprising is the fact that vector based methods are best on 18 of the problems (although this ignores both COTE and $\hbox {DTW}_F$) and elastic techniques do poorly on these problems (the best elastic method is 5.3% worse). This indicates the importance of choosing a representation, but does not help in choosing a method within a possible representation.

**Table 11:** A summary of the relationship between classes of algorithms

Best	#	Vector (%)	Elastic (%)	Interval (%)	Shapelet (%)	Dictionary (%)
Vector	18	0.00	$-$5.30	$-$3.67	$-$3.02	$-$5.34
Elastic	18	$-$9.52	0.00	$-$3.87	$-$4.86	$-$3.41
Interval	8	$-$4.77	$-$3.38	0.00	$-$2.17	$-$5.79
Shapelet	28	$-$9.69	$-$6.40	$-$5.54	0.00	$-$3.69
Dictionary	13	$-$14.98	$-$5.79	$-$6.47	$-$3.58	0.00

All problems are grouped by the type of algorithm which has the highest accuracy. Each table entry is the average difference in accuracy of the average of the best performing algorithms of the best in each category. So, for example, the best of the shapelet approaches (ST, LS and FS) is on average 3.58% less accurate than the dictionary approaches on problems where the dictionary approach is the most accurate overall

### Whole series methods

Of the three distance based approaches we evaluated (TWE, WDTW and MSM), MSM is the highest rank (9$\mathrm{th}$) and is the only one significantly better than both benchmarks. WDTW (ranked 14$\mathrm{th}$) is better than DTW but not RotF. This conclusion contradicts the results in Lines and Bagnall (2015) which found no difference between all the elastic algorithms and DTW. This is because whilst there is a significant improvement, the benefit is small, and undetectable without resampling. MSM is under 2% on average better than DTW and RotF. The average of average differences in accuracy between WDTW and DTW is only 0.2%.

In line with published results, two of the difference based classifiers, CID and $\hbox {DTD}_C$ are significantly better than DTW, but the mean improvement is very small (under 1%). None of the three difference based approaches are significantly different to RotF. We believe this highlights an over-reliance on DTW as a benchmark. Methods that provide a small improvement to DTW may just be making it a little better on problems where DTW is not suitable. We should also note that it is possible that simple improvements could improve the difference based classifiers. In line with the original description we set the CID warping window as the optimal for DTW. Setting the window to optimise the CID distance instead might well improve performance. Similarly, $\hbox {DD}_{\textit{DTW}}$ and $\hbox {DTD}_C$ use full window DTW and could be more accurate with windowed DTW or WDTW.

**Fig. 12:**
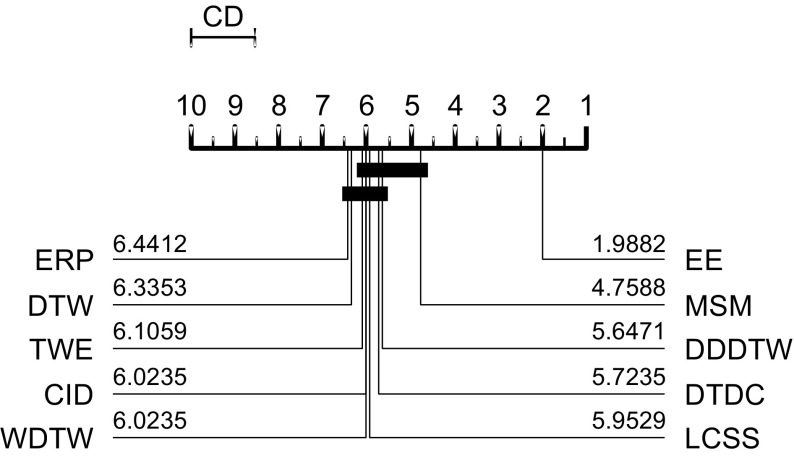
Critical difference diagram for ten elastic distance measures. The elastic ensemble (EE) is significantly more accurate than its constituents

Suppose then that through domain knowledge we believe the whole series should be used but there may be some need for allowing for some elasticity in indexing. Which approach should we take? Figure 12 shows the relative performance of the ten elastic measures considered.

**Fig. 13:**
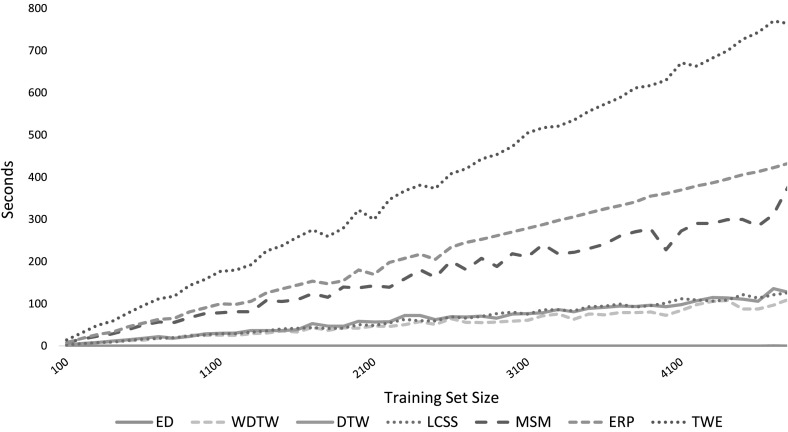
Time to classify ten test instances (averaged over 100 parameter options) for varying number of train instances of the problem StarlightCurves. The legend is ordered from fastest algorithm (ED) to slowest algorithm (TWE)

Clearly the answer is to use all of them and ensemble (the EE approach). However, this may not always be feasible due to computational limitations. Suppose timing is critical. Of all the measures used in Lines and Bagnall (2015), MSM and TWE were by far the slowest. Figure 13 shows the average time taken to classify 10 test instances of StarlightCurves using 7 different distance measures with a 1-NN classifier. DD, CID and DTD are omitted for clarity because they are simply two or three repetitions of DTW.

ED is of course much faster, but it is significantly less accurate. There is little to choose between DTW, WDTW and LCSS. They are on average approximately 1500 times slower than ED. ERP and MSM are significantly slower, taking on average 4500 and 5700 times longer than ED respectively. Slowest of all is TWE, which is over 10,000 times slower than ED on this dataset. We have not made a huge effort to optimise the efficiency of these algorithms. ED, DTW, WDTW, and MSM all have an early abandon. LCSS, ERP and TWE do not easily lend themselves to this approach. Our conclusion from Figs. 12 and 13 is that if a single measure is required it should be a choice between DTW and MSM, with MSM preferred if there is time because of its better average performance. It would be beneficial to investigate ways of speeding up MSM through better early abandoning. It is also useful to examine how good a choice we could make between MSM and DTW using a Texas sharpshooter plot (first described in Batista et al. 2014). These address the question of whether we could make the correct decision between two classifiers by plotting the ratio of train accuracies against test accuracies over multiple problems.

**Fig. 14:**
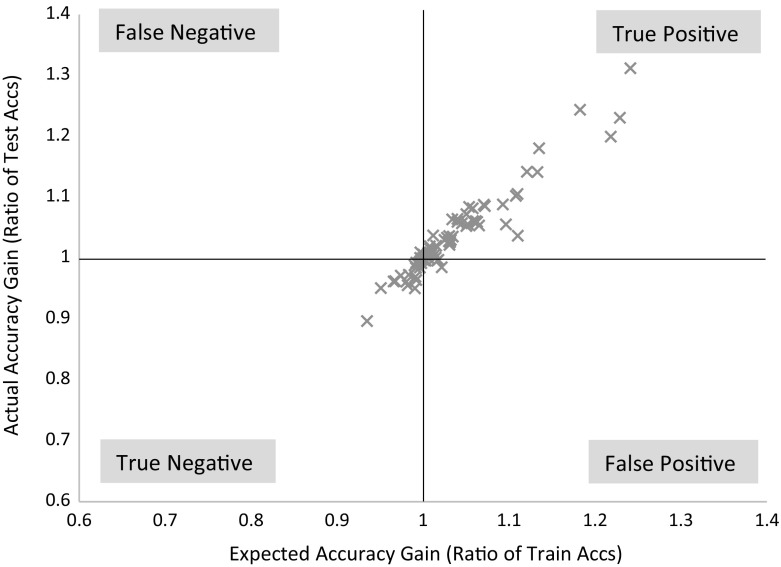
Texas sharp shooter plot for MSM against DTW. The *top right* quadrant contains the problems where both the train and test accuracy for MSM is higher than DTW

Figure 14 shows the Texas sharpshooter plot for MSM against DTW. We use the train and test accuracies of 85 datasets averaged over 100 repetitions. On 53 of the problems the greater train accuracy of MSM translates into better test accuracy. On 15 data sets, we would correctly predict DTW performs better. We only made an incorrect prediction in 20% of datasets. This suggests that an accurate decision between DTW and MSM can be made.

### Interval based classifiers

**Fig. 15:**
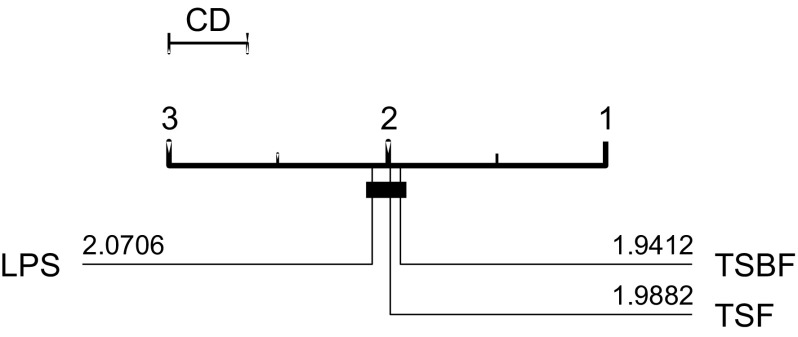
Critical difference diagram for three interval based techniques. There is no significant difference between them

The interval based approaches, TSF, TSBF and LPS, are all significantly better than both the benchmarks. This gives clear support to the idea of interval based TSC. Figure 15 shows there is no significant difference between the three approaches we have evaluated.

It is possible the fact TSBF and LPS do not outperform TSF is caused by our implementation of these complex algorithms. Variants may perform better. However, based on the evidence of this study, we conclude that TSF is the best interval approach to use because of its simplicity and speed, but that TSBF is a viable alternative. Although LPS is interval based, it is based on autocorrelation characteristics and may have desirable characteristics for some problems.

### Dictionary based classifiers

The results for window based dictionary classifiers are confusing. SAXVSM and BOP are significantly worse than the benchmarks and ranked 18$\mathrm{th}$ and 19$\mathrm{th}$ overall respectively. This would seem to suggest there is little merit in dictionary transformations for TSC. However, the BOSS ensemble is one the most accurate classifiers we tested. It is significantly better than both benchmarks and is ranked third overall. All three approaches involve taking windows along the series, approximating and discretising the resulting subseries then forming a distribution of word counts, so the difference in performance is puzzling. There are four key differences between BOP/SAXVSM and BOSS.

1. *Ensemble* The BOSS ensemble retains all classifiers with training accuracy within 92% of the best.
2. *Window approximation* BOP and SAXVSM use PAA to approximate whereas BOSS uses the truncated DFT.
3. *Word discretisation* BOSS employs a data driven discretisation rather than fixed break points used by BOP and SAXVSM.
4. *Distance measure* BOSS uses a non symmetric distance function that ignores words missing from the first series as opposed to BOP which uses Euclidean distance and SAXVSM that uses cosine difference.

In order to gauge the relative importance of each of these four factors, we re-ran four sets of experiments with one of the above features turned off (i.e. BOSS used the method employed in BOP instead). *A priori* we thought that one or possibly two of the four features would explain the improvement. However, each change resulted in significantly worse accuracy in comparison to the full BOSS ensemble. This makes us conclude that all four design features are necessary for BOSS. The largest change was found by using the single best BOSS classifier rather than an ensemble. This reduced the accuracy on average by over 5%. This re-enforces our general point about the importance of ensembling to maximize accuracy, particularly on problems with small train sets. Almost as striking was the difference in using PAA against the truncated DFT. PAA reduced the average accuracy of the BOSS ensemble by over 5% also. This is surprising, since previous studies have shown little difference in the approximation power of PAA and DFT. The bespoke non-symmetric distance measure made BOSS more accurate by 3% on average compared to Euclidean distance. This highlights the systemic problems of balancing loss of information through discretisation against lack of discrimination due to the sparseness of the histograms. The least amount of improvment came from using MCB rather than fixed interval discretisation, although the effect was still significant.

### Shapelet based classifiers

FS is the least accurate classifier we tested and is significantly worse than the benchmarks. In line with the original shapelet research (Ye and Keogh 2011), FS is used in conjunction with a decision tree. The key component of FS is the rapid discovery of shapelets, and there is no reason FS could not be imbedded in the shapelet transform. We are currently investigating whether using the FS speed up makes any difference to the accuracy of ST.

LS is not significantly better than Rotation Forest and is only marginally better than DTW. We have put considerable effort into debugging LS and have been in correspondence with the author, who has edited and adapted our code. However, it is a complex algorithm, so it is possible bugs remain. Gradient descent methods such as LS are known to be sensitive to train set size, and the large number of relatively small training set sizes of the TSC archive datasets could hinder LS. Analysis of the aggregated results indicates that a more important factor is that LS performs poorly on multiple class problems. It uses a logistic model for each class, hence the parameter space grows for each extra class. Examination of the results for repetitions on individual problems indicates that on occasional runs LS converges to very poor solutions. This is always a risk with gradient descent and it will influence average performance.

ST has exceeded our expectations. It is significantly better than both benchmarks and is the second most accurate classifier overall, significantly better than six of the other eight classifiers that beat both benchmarks. It is significantly better than FS and LS. The changes proposed in Bostrom and Bagnall (2015) have not only made it much faster, but have also increased accuracy. Primary amongst these changes is balancing the number of shapelets per class and using a one-vs-many shapelet quality measure. However, ST is the slowest of all the algorithms we assessed and there is scope to investigate methods of increasing the speed without compromising accuracy. For example, it is likely that the train set for shapelet search could be condensed without loss of accuracy, because by definition shapelets appear in a large number of cases of the same class.

### Combining classifiers and representations

We believe that combining classifiers is the easiest way to get improvement for TSC without the direct application of domain knowledge. Our results show that ensembling over a single representation is very effective; the top seven classifiers are all ensembles. It seems highly likely the other classifiers would benefit from a similar approach. One of the key ensemble design decisions is promoting diversity without compromising accuracy. TSF, TSBF and LPS do this through the standard approach of sampling the same attribute space. BOSS ensembles identical classifiers with different parameter settings. ST and EE engender diversity though classifier heterogeneity. Employing different base classifiers in an ensemble is relatively unusual, and these results would suggest that it might be employed more often. However, for TSC the best results come from combining classifiers on different representations. COTE is significantly better than all other classifiers we have evaluated. It promotes diversity through employing different classifiers on a range of transformations/data representations and weighting by a training set accuracy estimate. Its simplicity is its strength. These experiments suggest COTE may be even more accurate if it were to assimilate BOSS and an interval based approach.

$\hbox {DTW}_F$ also did well (ranked 5$\mathrm{th}$). Including SAX features significantly improves $\hbox {DTW}_F$. This surprised us, and strengthens the case for combining representations. Without SAX histograms, the $\hbox {DTW}_F$ approach of using distances as features is not significantly better than DTW. Conversely, the BOP results show us that the SAX histograms on their own produce a poor classifier. Our conjecture is that the DTW features are compensating for the datasets that BOP does poorly on, whilst gaining from those it does well at. This supports the argument for combining features from different representations.

## A closer look at specific problems

In Sect. 2 we made qualitative arguments for scenarios for when each algorithm type would perform better, and in Sect. 5 we examined overall variation in performance between classes of algorithms. It is also desirable to look at some specific problems to help understand variation in performance. Our main focus has been on assessing algorithms by rank. This is the best way of mitigating against differences in the underlying Bayes error, but also may lead to the suspicion that the actual differences in accuracy are tiny. We examine the results for datasets with the widest gap in accuracy to help offer insights into the strengths and weaknesses of the algorithms.

### ToeSegmentation1 and ToeSegmentation2

These two data sets are originally from the CMU Graphics Lab Motion Capture Database^1^ and were formatted for classification in Ye and Keogh (2011). The data are two class problems with classes “Walk Normally” and “Walk Abnormally” taken from the left and right toe. The original series were of unequal length. The repository data have been truncated to the shortest series length (277 for ToeSegmentation1 and 343 for ToeSegmentation2). The series were subsequently normalised.

**Fig. 16:**
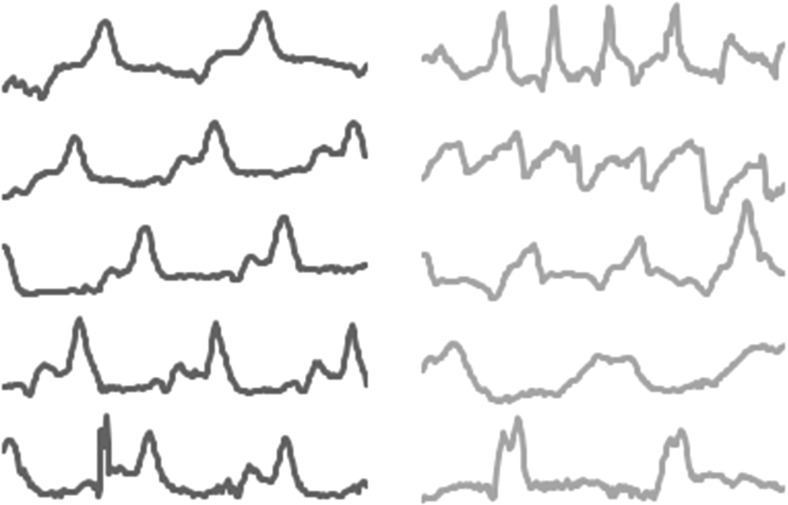
Example series from ToeSegmentation1, *left* is class 1 (normal walking) and *right* is class 2 (abnormal walking)

Figure 16 shows example series from both classes of ToeSegmentation1. Table 12 shows the mean accuracy of all 20 classifiers on this problem. Both problems have over 30% difference in accuracy between the best and the worst algorithm. The benchmark classifiers RotF is poor at these problems (accuracy of 57.8 and 64.6% respectively), because the peaks are not alligned in time. DTW is a little better (accuracy of 72.2 and 85.09%), but it is confounded by the fact the number of peaks (equating to strides) differ within each class. Of the interval classifiers, TSF is no better than DTW, but TSBF and LPS do better. The internal classification stage of these algorithms goes some way to mitigating the problems encountered by the whole series approaches. However, the best methods for this problem are dictionary based and shapelet based algorithms. The discriminatory features are the shapes of the peaks rather than the shape of the whole series. ST is the most accurate on ToeSegmentation1, BOSS the best on ToeSegmentation2 and COTE is ranked second and third. The hybrid $\hbox {DTW}_F$ also performs well. These datasets emphasise the importance of using the correct family of algorithms, or failing that, the importance of using techniques that combines representations.

### LargeKitchenAppliances

**Fig. 17:**
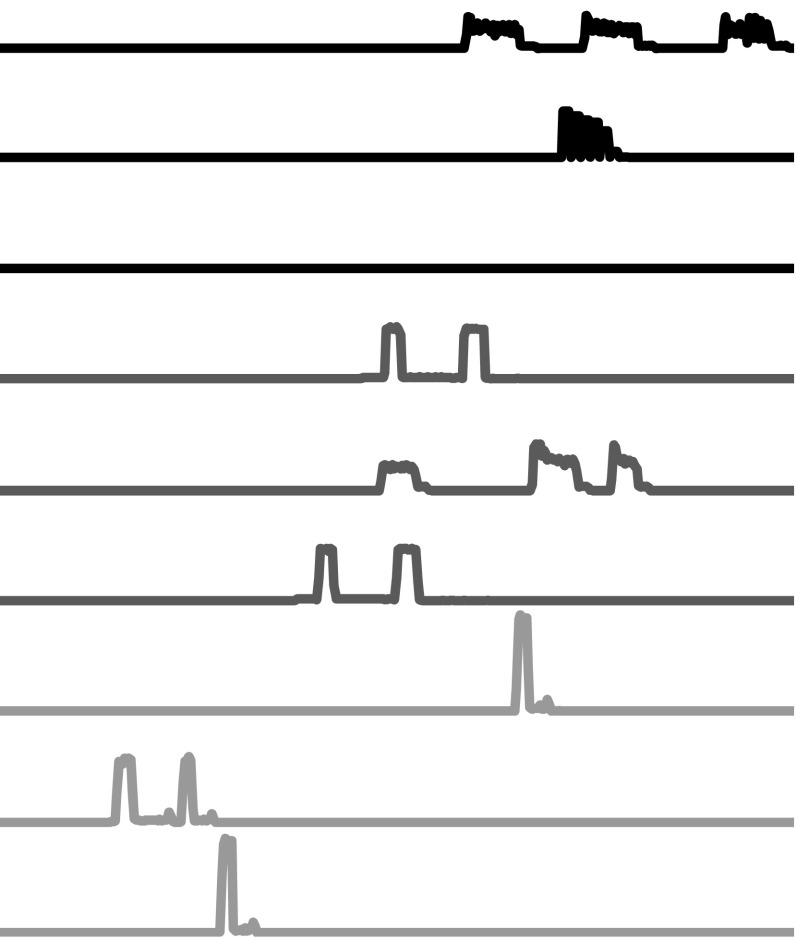
Example series from LargeKitchenApplicances. The *top three* are Washing Machine (class 3), the *middle three* Tumble Dryers (class 2) and the *bottom three* Dishwashers (class 1)

LargeKitchenAppliances is an electric device classification problem derived from the Powering the Nation study (Energy Saving Trust 2012) and first introduced in Lines and Bagnall (2015). Each series is length 720 (24 h of readings taken every 2 min), there are three classes (Washing Machine, Tumble Dryer and Dishwasher) and a 750 cases split evenly between train and test. The data is characterised by periods of inactivity followed by spikes when the device is in use (see Fig. 17).

**Table 12:** Results of all algorithms on ToeSegmentation1 and ToeSegmentation2

Algorithm	ToeSegmentation1	ToeSegmentation2
ST	**95.40%**	94.72%
LS	93.43%	94.26%
COTE	93.37%	95.15%
BOSS	92.88%	**95.97%**
SAXVSM	92.79%	92.08%
BoP	92.62%	91.17%
$\hbox {DTW}_F$	92.20%	90.38%
FS	90.41%	87.28%
TSBF	85.82%	88.58%
LPS	84.12%	92.64%
MSM	82.13%	89.52%
TWE	79.59%	88.85%
EE	78.76%	90.70%
$\hbox {DD}_{\textit{DTW}}$	74.18%	82.80%
$\hbox {DTD}_C$	72.90%	82.58%
WDTW	72.79%	86.22%
DTW	72.20%	85.09%
$\hbox {CID}_{\textit{DTW}}$	71.80%	84.39%
TSF	66.10%	78.24%
ED	61.20%	78.10%
RotF	57.80%	64.60%

Bold values indicate the best overall algorithm for each problem (i.e. ST is best at Toe1, Boss at Toe2)

There is a 40% difference in accuracy between the best and the worst classifier. Vector based algorithms such as RotationForest obtain accuracies around 60%. This is better than random guessing, because they are capturing the fact that devices might well be used at the same time, but this is only a weak feature for classification. Devices can and are used at different times within and between households. Interval based methods do slightly better (65–70%) because they are able to compensate for the large blank periods (i.e. high number of redundant features), but are still confounded by the time independence. Elastic measures are able to compensate for time shift and are more accurate still (75–80% range). However, the fact that all devices can and are used a varying number of times per day means full series elastic measures are of limited use. Dictionary techniques can overcome this somewhat (accuracy from 80 to 85%), but fail to do better because they lose granularity and best discriminatory features are not repeated patterns but unique subseries. The shapelet methods are best or this problem (90–95% accuracy) for this very reason. COTE does not improve on ST, indicating that this dataset does not contain independent features in the different domains.

### Adiac

The Automatic Diatom Identification and Classification (ADIAC) project was a pilot study concerning automatic identification of diatoms (unicellular algae) on the basis of images (Jalba et al. 2004). The outlines are extracted from thresholded images, but we do not have full details of the preprocessing. The series are 176 long. One defining characteristic of Adiac is there are 37 classes, each representing a different species of diatom.

The vector based methods do relatively well at this problem. SVM and RotF average around 75% accuracy. This contrasts with the elastic measures, which are all in the range 60–65%. This suggests that the images have been aligned and length normalised in preprocessing. Interval, dictionary and shapelet methods are all about the same as RotF at around 75% accuracy. Given the very different nature of these algorithms, it seems likely that different diatoms have different discriminatory features and hence require different representations. This hypothesis is supported by the fact that COTE is significantly more accurate than all other approaches (81% accuracy). If we had instigated this project these results would guide our area of research towards a detailed analysis of the contigency tables of each algorithm to better understand where the variation lies.

## Conclusions

The primary goal of this series of benchmark experiments is to promote reproducible research and provide a common framework for future work in this area. We view data mining as a practical area of research, and our central motivation is to find techniques that work. Received wisdom is that DTW is hard to beat. Our results confirm this to a degree (7 out of 19 algorithms fail to do so), but recent advances show it is not impossible.

Overall, our results indicate that COTE is, on average, clearly superior to other published techniques. It is on average 8% more accurate than DTW. However, COTE is a starting point rather than a final solution. Firstly, the no free lunch theorem leads us to believe that no classifier will dominate all others. The research issues of most interest are what types of algorithm work best on what types of problem and can we tell *a priori* which algorithm will be best for a specific problem. Secondly, COTE is hugely computationally intensive. It is trivial to parallelise, but its run time complexity is bounded by the Shapelet Transform, which is $O(n^2m^4)$ and the parameter searches for the elastic distance measures, some of which are $O(n^3)$. ST and EE are also trivial to distribute, but there is a limit to the number of processors anyone can run in parallel. An algorithm that is faster than COTE but not significantly less accurate would be a genuine advance in the field. Finally, we are only looking at a very restricted type of problem. We have not considered multi-dimensional, streaming, windowed, long series or semi-supervised TSC, to name but a few variants. Each of these subproblems would benefit from a comprehensive experimental analysis of recently proposed techniques.

There are numerous weaknesses in our study which we freely acknowledge. The selection of data is ad-hoc and many of the datasets are related to each other. We hope to overcome this by increasing the archive size so that it may then be sensibly sampled by problem type. We are constantly looking for new areas of application and we will include any new data sets that are donated in an ongoing evaluation. We stress that accuracy is not the only consideration when assessing a TSC algorithm. Time and space efficiency are often of equal or greater concern. However, if the only metric used to support a new TSC is accuracy on these test problems, then we believe that evaluation should be transparent and comparable to the results we have made available. If a proposed algorithm is not more accurate than those we have evaluated, then some other case for the algorithm must be made.

Furthermore, we have not optimised the algorithms or necessarily been fair to them all. For example, we have fixed the number of parameter contributions for each to 100. This could be considered unfair to the faster algorithms. Ideally we would like to give each algorithm a fixed amount of computational time, but we found this impractical. Our code is not necessarily optimised for all algorithms and some are distributed.

Many of the algorithms are stochastic, and hill climbers such as LS may well improve if we included number of restarts as a parameter. We did not do this because it introduces another level of cross validation and we considered that, given the fixed number of parameter evaluations, it would be more productive to do model selection within the specific parameter space of each algorithm. These were pragmatic decisions, and we will happily evaluate anyone else’s new or refined algorithm if it is implemented as a WEKA classifier (with all model selection performed in the method buildClassifier) and if it is computationally feasible. If we are given permission we will release any results we can verify through the associated website. We also acknowledge that despite our best efforts and communication with the original authors, our implementations may not be bug free. We will examine any alterations submitted to us and if an error is found, rerun that classifier.

For those looking to build a predictive model for a new problem we would recommend starting with DTW, RandF and RotF as a basic sanity check and benchmark. We have made little effort to perform model selection for the forest approaches because it is generally accepted they are robust to parameter settings, but some consideration of forest size and tree parameters may yield improvements. However, our conclusion is that using COTE will probably give you the most accurate model. If a simpler approach is needed and the discriminatory features are likely to be embedded in subseries, then we would recommend using TSF or ST if the features are in the time domain (depending on whether they are phase dependent or not) or BOSS if they are in the frequency domain. If a whole series elastic measure seems appropriate, then using EE is likely to lead to better predictions than using just DTW.

## Footnotes

1 CMU Graphics Lab Motion Capture Database http://mocap.cs.cmu.edu/.
